# Carbonic anhydrase XII mediates the survival and prometastatic functions of macrophages in human hepatocellular carcinoma

**DOI:** 10.1172/JCI153110

**Published:** 2022-04-01

**Authors:** Wan-Ru Ning, Da Jiang, Xing-Chen Liu, Yu-Fan Huang, Zhi-Peng Peng, Ze-Zhou Jiang, Tiebang Kang, Shi-Mei Zhuang, Yan Wu, Limin Zheng

**Affiliations:** 1 Guangdong Province Key Laboratory of Pharmaceutical Functional Genes, MOE Key Laboratory of Gene Function and Regulation, School of Life Sciences, Sun Yat-sen University, Guangzhou, China.; 2 State Key Laboratory of Oncology in Southern China, Collaborative Innovation Center for Cancer Medicine, Sun Yat-sen University Cancer Center, Guangzhou, China.

**Keywords:** Immunology, Metabolism, Cancer immunotherapy, Liver cancer, Monocytes

## Abstract

Macrophages constitute a major immune component in tumor tissues, but how these cells adapt to and survive in the nutrient-depleted and lactic acid–induced acidic tumor microenvironments is not yet fully understood. Here, we found that levels of carbonic anhydrase XII (CA12) expression were significantly and selectively upregulated on macrophages in human hepatocellular carcinoma (HCC). Transient glycolytic activation of peritumoral monocytes induced sustained expression of CA12 on tumor-infiltrating macrophages via autocrine cytokines and HIF1**α** pathways. On the one hand, CA12 mediated the survival of macrophages in relatively acidic tumor microenvironments, while on the other hand, it induced macrophage production of large amounts of C-C motif chemokine ligand 8 (CCL8), which enhanced cancer cell epithelial-mesenchymal transition (EMT) and facilitated tumor metastasis. Consistently, the accumulation of CA12^+^ macrophages in tumor tissues was associated with increased tumor metastatic potential and reduced survival of patients with HCC. Selective targeting of tumor-infiltrating macrophages with a CA12 inhibitor reduced tumor growth in mice and was sufficient to synergistically enhance the therapeutic efficacy of immune-checkpoint blockade. We suggest that CA12 activity is a previously unappreciated mechanism regulating the accumulation and functions of macrophages in tumor microenvironments and therefore represents a selective vulnerability that could be exploited in future designs for antitumor immunotherapeutic strategies.

## Graphical abstract

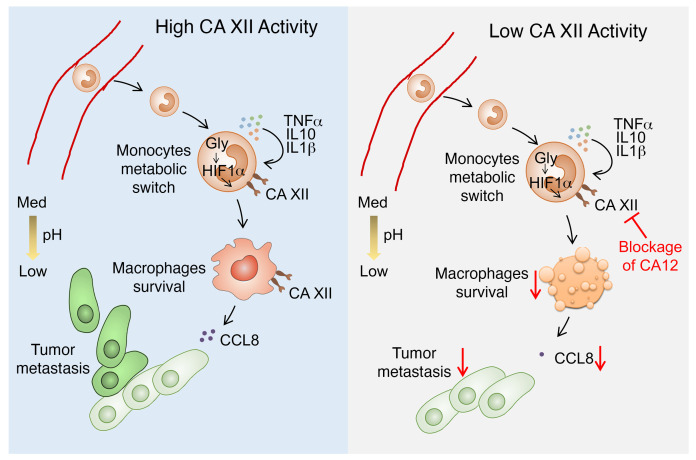

## Introduction

Macrophages constitute a major component in most human solid tumors and perform various functions according to local environmental cues (1–4). Under the influence of different tumor tissues or distinct regional environments within a tumor, macrophages can produce cytokines, chemokines, MMPs, and many other factors to facilitate tumor metastasis, angiogenesis, metabolism, immune evasion, and even therapeutic resistance (5–8). In light of their important roles in almost every step of tumor progression, macrophages have been of great interest as a target in the field of cancer immunotherapy (9–12). However, neither depletion of macrophages nor reprogramming of their functions has yielded the expected satisfactory results, and the diversity or lack of tumor-specific markers for macrophages might pose the largest barrier to therapeutically targeting these cells.

Acidosis is a common characteristic exhibited by most solid tumors. The buildup of lactic acid created by proliferative cancer cells constitutes an adverse environment for the survival of macrophages, a majority of which are recruited and differentiated from peripheral monocytes (13–15). However, the de facto abundance and accumulation of macrophages in tumor tissues suggest that these cells might adopt unique mechanisms to adjust to toxic microenvironments, thus possibly exposing a vulnerability that could be exploited by an immunotherapy aiming to target these cells.

Carbonic anhydrases (CAs) are well-known zinc metalloproteins involved in the catalysis of a very simple but essential physiological reaction: carbon dioxide hydration to bicarbonate and a proton (12). Among the 15 isoforms known in humans, 2 cell-surface CA isoforms, namely, CA9 (or CA IX, almost exclusively associated with tumors) and CA12 (or CA XII, upregulated in some tumor types), are involved in tumorigenesis (16, 17). The crucial roles of CA9 and CA12 in intracellular pH maintenance represent the means by which cancer cells adapt to the toxic conditions of the extracellular milieu (18–20). Therefore, targeting tumor-associated CAs, especially CA9, is now considered a pertinent approach for the development of new cancer therapeutics to treat hypoxic tumors (21). However, the expression of CA9 and CA12 is not universal across tumor types or individual patients, and until now, the major focus has been only their roles in regulating cancer cell survival (22–24). The possible functions of CAs in mediating the adaptation and accumulation of large amounts of macrophages in tumor microenvironments are largely unknown.

In the current study, we found that levels of CA12 expression were substantially upregulated in tumor tissues compared with in paired nontumor liver tissues from hepatocellular carcinoma (HCC) patients and that CA12 was preferentially expressed by monocytes and macrophages. A transient metabolic switch induced the sustained expression of CA12 on macrophages via HIF1α- and autocrine cytokine-dependent pathways, and upregulated CA12 not only mediated the survival of macrophages in lactic acid–induced acidic culture conditions, but also directly facilitated HCC tumor metastasis and progression by inducing macrophage production of C-C motif chemokine ligand 8 (CCL8). CA12 thus represents a selective marker of HCC-infiltrating macrophages with both diagnostic potential and therapeutic potential.

## Results

### CA12 is selectively upregulated on tumor-infiltrating monocytes and macrophages and correlates with disease progression.

**Figure 1:**
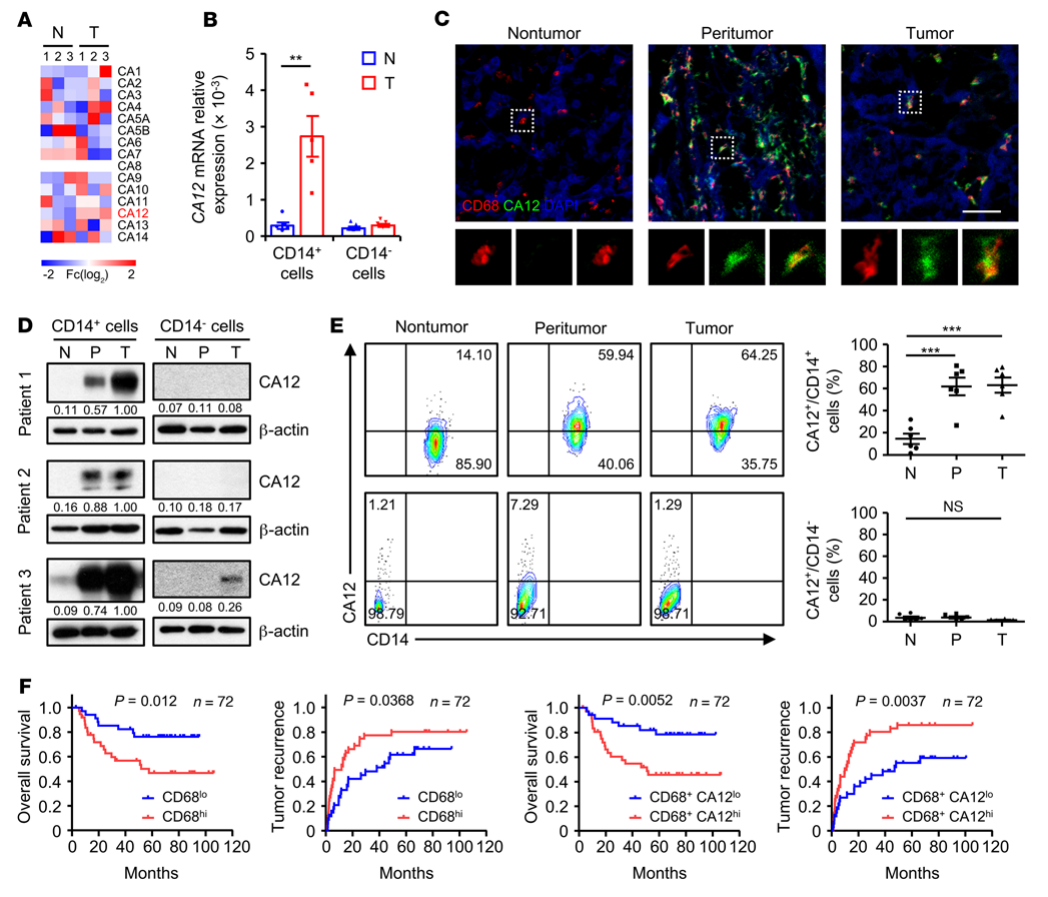
CA12 is selectively upregulated on tumor-infiltrating monocytes and macrophages and correlates with disease progression. (**A**) CD14^+^ cells were purified from paired nontumor (N) and tumor tissues (T) from 3 patients with HCC. The levels of CD14^+^ cell αCA family gene expression were quantified by qPCR. (**B**) CD14^+^ and CD14^–^ leukocytes were purified from paired nontumor and tumor tissues from HCC patients. Levels of *CA12* expression in these cells were quantified by qPCR (*n* = 5). (**C**) Frozen sections of HCC samples were stained with an anti-human CD68 antibody (red), an anti-human CA12 antibody (green), and DAPI (blue). The colocalization and distribution of cell signals were analyzed by confocal microscopy. Scale bar: 50 μm. One out of five representative micrographs from 5 independent experiments is shown. (**D**) CD14^+^ and CD14^–^ leukocytes were purified from paired nontumor, peritumor (P), and tumor tissues from HCC patients. The levels of CA12 expression in these cells were determined by immunoblotting (*n* = 3). (**E**) Fresh leukocytes were isolated from paired nontumor, peritumor, and tumor tissues from HCC patients. Levels of CA12 expression on CD14^+^ and CD14^–^ leukocytes were determined by flow cytometry (*n* = 6). (**F**) Seventy-two HCC patients who underwent curative resection with follow-up data were divided into 2 groups according to the median value of CD68^+^ or CD68^+^CA12^+^ cell density in tumor tissues (CD68^+^ cells: low, ≤391 cells/mm^2^ [*n* = 35]; high, >391 cells/mm^2^ [*n* = 37]; CD68^+^CA12^+^ cells: low, ≤278 cells/mm^2^ [*n* = 35]; high, >278 cells/mm^2^ [*n* = 37]). The OS and TR of these patients were analyzed via the Kaplan-Meier method and log-rank test. Results shown in **B** and **E** are represented as mean ± SEM. *P* values were obtained by paired 2-tailed Student’s *t* test (**B**), 1-way ANOVA (**E**), or log-rank test (**F**). **P < 0.01; ***P < 0.001.

By comparing genes differentially expressed by monocytes purified from tumor and paired nontumor liver tissues of HCC patients, we found that, among all αCA family genes, CA12 was the most markedly upregulated gene in tumor-infiltrating monocytes compared with those from nontumor liver tissues (Figure 1A). Through enlarging patient samples, we verified the increase in the *CA12* mRNA level in tumor-infiltrating monocytes and found that other CD14^–^ cellular components did not express CA12 in either tumor tissues or nontumor liver tissues (Figure 1B).

To confirm the expression of CA12 in tumor-infiltrating monocytes and macrophages in situ, serial sections of HCC tissue samples were double stained with anti-human CD68 and anti-human CA12 antibodies. As shown in Figure 1C and Supplemental Figure 1 (supplemental material available online with this article; https://doi.org/10.1172/JCI153110DS1), CA12 was significantly and specifically upregulated in peritumoral and tumor-infiltrating CD68^+^ cells compared with their nontumor counterparts, whereas a weak signal or no substantial signal for CA12 was detected for other cellular components in tumor, peritumor, and nontumor liver tissues. The significant upregulation of CA12 in and on tumor-purified monocytes was further confirmed by immunoblotting and flow cytometry analysis, which also showed that all the other CD14^–^ cellular components expressed none to very low levels of CA12 regardless of their tissue area of origin (Figure 1, D and E). Moreover, patients with high CA12 expression exhibited significantly more CD68^+^ cell infiltration than those with low CA12 expression (Supplemental Figure 2A), and immunofluorescence staining of frozen sections of HCC tissue samples showed that most CA12^+^ cells in tumor tissues were also positive for the M2 markers CD204 and CD206 (Supplemental Figure 2, B and C).

To explore the potential role of CA12 expression by monocytes and macrophages in disease progression, we divided HCC patients who had undergone curative resection with follow-up data into 2 groups according to the median value of CD68^+^ or CD68^+^ CA12^+^ cell density in tumor tissues. As shown in Figure 1F, high levels of both CD68^+^ and CD68^+^CA12^+^ cell infiltration in tumor regions indicated worse patient survival, with the CD68^+^CA12^+^ groups exhibiting better prognostic values than the CD68^+^ groups (CD68^+^: *P* = 0.012 for overall survival [OS], *P* = 0.0368 for tumor recurrence [TR]; CD68^+^CA12^+^: *P* = 0.0052 for OS, *P* = 0.0037 for TR). Moreover, the density of CD68^+^CA12^+^ cells in tumor tissues could serve as an independent prognostic factor for both the OS and TR of HCC patients (Supplemental Table 1). The above results suggested that the selective and significant upregulation of CA12 on tumor-infiltrating monocytes and macrophages was associated with the accumulation of these cells and might facilitate disease progression in human HCC.

### CA12 expression is positively correlated with glycolytic switch in tumor-infiltrating monocytes and macrophages.

**Figure 2:**
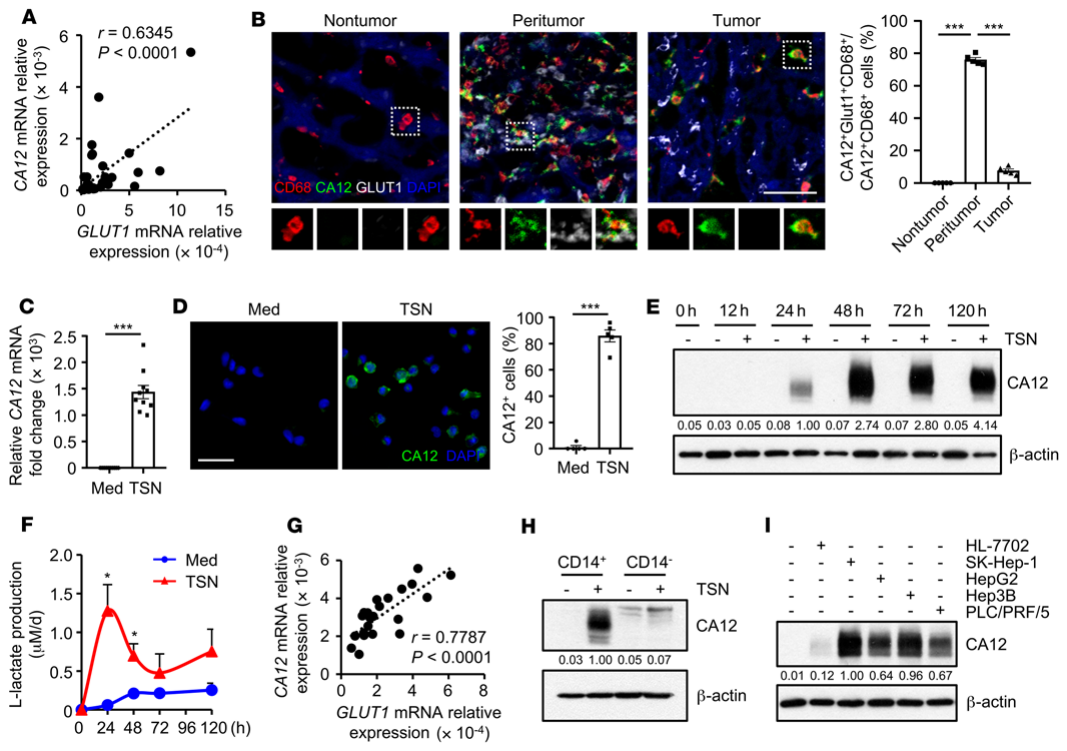
CA12 expression is positively correlated with glycolytic switch in tumor-infiltrating monocytes and macrophages. (**A**) CD14^+^ cells were purified from tumor tissues from 41 patients with HCC. Correlations between the mRNA levels of *GLUT1* and *CA12* in these cells were analyzed. (**B**) Frozen sections of HCC samples were stained with an anti-human CD68 antibody (red), an anti-human CA12 antibody (green), an anti-human GLUT1 antibody (white), and DAPI (blue). The colocalization of cell signals was analyzed by confocal microscopy. Scale bar: 50 μm. *n* = 5. (**C**–**I**) CD14^+^ and CD14^–^ leukocytes were purified from the peripheral blood of healthy donors. (**C** and **D**) CD14^+^ cells were left untreated (Med) or treated with supernatants from HepG2 cells (TSN) for 24 hours. Levels of CA12 expression were determined by qPCR (**C**) (*n* = 10) and confocal microscopy (**D**) (*n* = 5; CA12: green; DAPI: blue). Scale bar: 50 μm. (**E** and **F**) CD14^+^ cells were left untreated or treated with HepG2 TSN for the indicated times. Levels of CA12 expression were determined by immunoblotting (**E**) (*n* = 5), and levels of lactate production were measured with a lactate assay kit (**F**) (*n* = 4). (**G**) CD14^+^ cells were treated with HepG2 TSN for 24 hours, and correlations between mRNA levels of *GLUT1* and *CA12* in these cells were analyzed (*n* = 22). (**H**) CD14^+^ and CD14^–^ leukocytes were left untreated or treated with HepG2 TSN for 24 hours, and levels of CA12 expression were determined by immunoblotting (*n* = 3). (**I**) CD14^+^ cells were left untreated or treated with TSN from different cell lines for 48 hours, and levels of CA12 expression in the cells were determined by immunoblotting (*n* = 3). Results shown in **B**–**D** and **F** are represented as mean ± SEM. *P* values were obtained by Pearson’s correlation and linear regression analysis (**A** and **G**), 2-way ANOVA (**B**), or 2-tailed Student’s *t* test (**C**, **D**, and **F**). *P < 0.05; ***P < 0.001.

Our previous study found that monocytes and macrophages selectively upregulated aerobic glycolysis in HCC tumors to adapt to their tissue-specific functions (25, 26). To explore whether such a metabolic switch might play a role in the induction of CA12 expression on these cells, we purified CD14^+^ cells from tumor tissues of HCC patients. As shown in Figure 2A, the expression levels of CA12 and glucose transporter *GLUT1* showed positive correlation in tumor purified CD14^+^ cells. Serial sections from HCC patients were then triple stained with antihuman CD68, antihuman GLUT1, and antihuman CA12 antibodies to confirm the colocalization of GLUT1 and CA12 on monocytes and macrophages in situ. Interestingly, while CD68^+^ macrophages expressed significant levels of both GLUT1 and CA12 in peritumoral HCC regions, they maintained high expression of only CA12, not GLUT1, in intratumoral tissues (Figure 2B). In comparison, CD68^+^ macrophages did not exhibit any positive staining for either GLUT1 or CA12 in paired nontumor liver tissues (Figure 2B). These data imply that tumor-derived factors might induce transient activation of glycolysis, but sustained upregulation of CA12, in HCC tumor–associated macrophages.

To study the potential mechanistic link between the glycolytic switch and CA12 expression, we established an in vitro model using CD14^+^ cells purified from the peripheral blood of healthy donors. These cells were treated with HepG2 hepatoma culture supernatants (tumor culture supernatants [TSN]) for 24 hours. TSN effectively induced the upregulation of both the mRNA and protein levels of CA12 in CD14^+^ cells in comparison with the medium control (Figure 2, C–E), and such upregulation could be sustained late after the differentiation of these cells into macrophages (Figure 2E). In contrast, TSN could also induce the upregulation of lactate production by monocytes, but the increase of lactate was transient, with a peak at approximately 24 hours after treatment (Figure 2F). Importantly, the mRNA expression levels of *CA12* and *GLUT1* showed positive correlation in TSN-treated CD14^+^ cells at early culture, similar to the correlation observed in their HCC tumor–purified counterparts (Figure 2G). The levels of CA12 expression in healthy donor peripheral blood–derived CD14^–^ cells were very low, whether treated with TSN or left untreated (Figure 2H), which was also consistent with clinical observations. Moreover, in addition to supernatants from HepG2 cells, TSN from other hepatoma cell lines (SK-Hep-1, Hep3B, and PLC/PRF/5), but not supernatants from the normal liver cell line HL-7702, could also induce the upregulation of CA12 in CD14^+^ cells (Figure 2I). Overall, we established an in vitro culture model to induce tumor-associated monocytes and macrophages with phenotypes similar to those of their counterparts in tumors in situ.

### Glycolysis induces CA12 upregulation in monocytes and macrophages via HIF1α and autocrine cytokine-dependent pathways.

**Figure 3:**
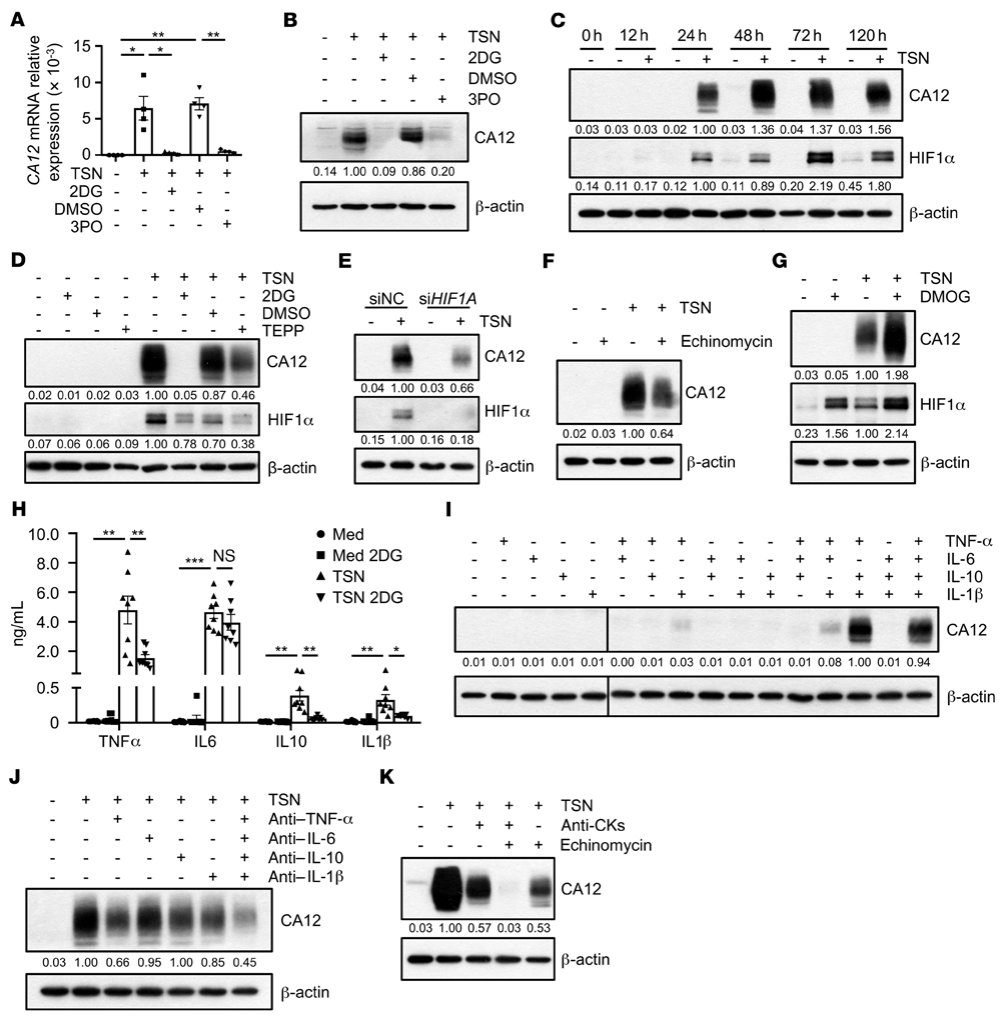
Glycolysis induces CA12 upregulation in monocytes and macrophages via HIF1α and autocrine cytokine-dependent pathways. CD14^+^ cells were purified from the peripheral blood of healthy donors. (**A** and **B**) Cells were left untreated or treated with TSN in the presence or absence of 2DG and 3PO. CA12 expression were determined by qPCR (**A**) and immunoblotting (**B**). *n* = 4. (**C**) Cells were left untreated or treated with TSN for the indicated times. Levels of CA12 and HIF1α were determined by immunoblotting (*n* = 5). (**D**) Cells were left untreated or treated with TSN in the presence or absence of DMSO, TEPP-46 (TEPP) or 2DG. CA12 and HIF1α expressions were determined by immunoblotting (*n* = 5). (**E**) Cells were transfected with siNC or si*HIF1A* before being treated with or without TSN. CA12 and HIF1α expressions were determined by immunoblotting (*n* = 4). (**F–H**) Cells were left untreated or treated with TSN in the presence or absence of echinomycin, DMOG or 2DG. CA12 and HIF1α expressions were determined by immunoblotting (**F** and **G**) (*n* = 4), and levels of cytokines production were measured by ELISA (**H**) (*n* = 8). (**I** and **J**) Cells were left untreated or treated with recombinant human cytokines or indicated neutralizing antibodies of cytokines, CA12 expression were determined by immunoblotting (*n* = 3). CA12 or β-actin lanes in **I** were run on the same gel, but were noncontiguous. (**K**) Cells were left untreated or treated with TSN in the presence or absence of anti-CKs (anti–TNF-α, anti–IL-10, and anti–IL-1β neutralizing antibodies) or/and echinomycin. CA12 expression were determined by immunoblotting (*n* = 3). Results shown in **A** and **H** are represented as mean ± SEM. *P* values were obtained by 1-way ANOVA (**A**) or 2-way ANOVA (**H**). *P < 0.05; **P < 0.01; ***P < 0.001.

To confirm the role of aerobic glycolysis in regulating CA12 expression in tumor-exposed monocytes and macrophages, CD14^+^ cells were purified from the peripheral blood of healthy donors and treated with HepG2 TSN in the presence or absence of the glycolysis inhibitor 2-deoxy-d-glucose (2DG) or the PFKFB3 (a key glycolytic enzyme) inhibitor 3PO. As shown in Figure 3, A and B, both 2DG and 3PO effectively attenuated the upregulation of CA12 mRNA and protein levels in tumor-treated monocytes. Given that glycolysis and HIF1α are highly interconnected pathways (27), we explored the possible role of HIF1α in mediating CA12 induction by glycolytic activation. While TSN could induce a sustained increase in HIF1α in CD14^+^ cells compared with control medium, both 2DG and TEPP, a PKM2 activator reported to inhibit the PKM2-induced stabilization of HIF1α (27), could substantially attenuate the TSN-induced upregulation of HIF1α and CA12 expression in these cells (Figure 3, C and D). Moreover, treatment with either si*HIF1A* or echinomycin, a competitive HIF1α inhibitor, abrogated TSN-induced CA12 upregulation in monocytes compared with control treatment (Figure 3, E and F), while dimethyloxallyl glycine (DMOG), an HIF1α inducer, acted synergistically with TSN to induce the upregulation of CA12 expression in these cells (Figure 3G).

Of note, inhibition of HIF1α could only partially attenuate the induction of CA12 by TSN, and DMOG alone could not induce the upregulation of CA12, indicating that additional mechanisms might be involved in the regulation of CA12 by glycolysis in monocytes. Since hepatoma TSN could induce significant production of cytokines such as TNF-α, IL-6, IL-10, and IL-1β in monocytes and this induction (with the exception of IL-6) could be effectively attenuated by treatment of these cells with the glycolysis inhibitor 2DG (Figure 3H), we hypothesized that some of the autocrine cytokines might also be involved in the regulation of CA12 expression by aerobic glycolysis in monocytes. As shown in Figure 3I, TNF-α, IL-10, and IL-1β mimicked the effects of TSN in inducing the upregulation of CA12 expression in monocytes, and consistently, anti–TNF-α, anti–IL-10, and anti–IL-1β antibodies attenuated the induction of CA12 in tumor-exposed monocytes (Figure 3J). In contrast, neither treatment with IL-6 nor blockade of IL-6 exerted any effects on CA12 expression in these cells (Figure 3, I and J). Importantly, treatment with echinomycin combined with anti-CKs (anti–TNF-α, anti–IL-10, and anti–IL-1β antibodies) completely attenuated TSN-induced CA12 upregulation in monocytes in comparison with either treatment alone (Figure 3K), confirming a synergistic effect between autocrine cytokines and the HIF1α-signaling pathway in regulating CA12 expression by these cells.

We went on to confirm the correlation among CA12, HIF1α, and cytokine expression in HCC tumor tissue–purified CD14^+^ cells. As shown in Supplemental Figure 3, A–E, mRNA levels of *CA12* exhibited positive correlations with those of *HIF1A*, *TNF-A*, *IL-10*, and *IL-1B*, but not those of *IL-6*, in tumor-derived monocytes. Together, the above data suggest that the tumor-triggered glycolytic switch in monocytes induced the activation of HIF1α and production of TNF-α, IL-10, and IL-1β in these cells, which in turn synergistically upregulated the expression of CA12 in the monocytes.

### CA12 mediates the survival of monocytes and macrophages in acidic microenvironments.

To explore the possible role of CA12 in tumor-associated monocytes and macrophages, we treated peripheral CD14^+^ cells with HepG2 TSN in the presence of negative control siRNA (siNC), CA12 siRNA (si*CA12*). As shown in Supplemental Figure 4A, while si*CA12* effectively abrogated TSN-induced CA12 expression in monocytes compared with the siNC control, it did not influence the TSN-induced production of cytokines (TNF-α, IL-6, IL-10, and IL-1β) or the TSN-induced upregulation of immune-related surface markers (PD-L1, HLA-DR, CD80, and CD86) in or on these cells (Supplemental Figure 4, B–D).

**Figure 4:**
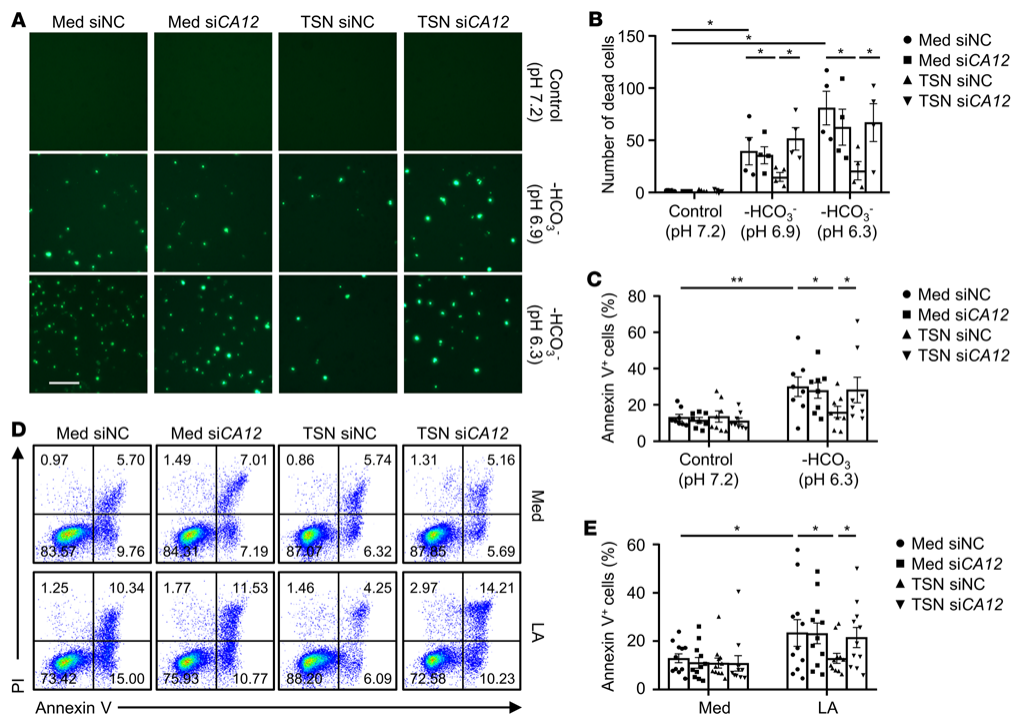
CA12 mediates the survival of monocytes and macrophages in acidic microenvironments. (**A**–**E**) CD14^+^ cells were purified from the peripheral blood of healthy donors. Cells were transfected with siNC or si*CA12* and then treated with or without HepG2 TSN for 48 hours before being exposed to pH 7.2 control medium (Med), pH 6.9 HCO_3_^–^-free medium, pH 6.3 HCO_3_^–^-free medium, or L-lactic acid (LA) (20 mM) for another 48 hours. (**A** and **B**) Dead cells were stained with SYTOX Green and analyzed by fluorescence microscopy (*n* = 4). One out of five representative graphs is shown in **A**. Scale bar: 50 μm. (**C**–**E**) Apoptosis of the cells was analyzed by flow cytometry. *n* = 8 (**C**); *n* = 11 (**D** and **E**). Results shown in **B**, **C**, and **E** are represented as mean ± SEM. *P* values were obtained by 1-way ANOVA. *P < 0.05; **P < 0.01.

Given that CA family members have been reported as playing important roles in buffering intracellular pH for cell survival in acidic conditions (18, 19), we aimed to elucidate whether the upregulated CA12 on tumor-associated monocytes and macrophages might mediate the survival of these cells in acidic tumor environments. As shown in Figure 4, A and B, the number of SYTOX Green^+^ dead monocytes was significantly increased in acidic culture conditions (HCO_3_^–^–free buffered pH 6.9 and 6.3 mediums) compared with normal control conditions (pH 7.2), and treatment with si*CA12* did not affect the survival of these cells. In contrast, acidic culture conditions did not markedly increase the number of SYTOX Green^+^ cells in tumor-exposed monocytes, but treatment with si*CA12* markedly upregulated the SYTOX Green^+^ cells in this treatment group. The prosurvival effect of CA12 on tumor-exposed monocytes was further confirmed through flow cytometry analysis, which showed that whether acidic conditions were induced with HCO_3_^–^–free medium (Figure 4C) or lactic acid (Figure 4, D and E), the acidic conditions could trigger apoptosis in normal monocytes, but not in their TSN-treated counterparts, and treatment with si*CA12* could significantly increase apoptosis in TSN-treated monocytes, but not in normal monocytes compared with their respective control cells. These results indicated that tumor-induced CA12 could mediate the survival of monocytes and macrophages in acidic microenvironments, which might partially explain the relatively abundant infiltration of this cell population observed in HCC tumor tissues.

### CA12 induces CCL8 production in tumor-associated monocytes and macrophages.

In addition to inflammatory cytokines and immune-related surface molecules, macrophages could regulate tumor progression via diverse mediators (28–30). To determine whether upregulated CA12 might directly influence other functions (in addition to survival) of tumor-associated monocytes and macrophages, we analyzed the correlations between the density of CA12^+^CD68^+^ cells in tumor tissues and the clinicopathological characteristics of HCC patients. As shown in Supplemental Table 2, levels of CA12^+^CD68^+^ cell infiltration were positively associated with the metastatic potential of tumor patients (*P* = 0.032).

**Figure 5:**
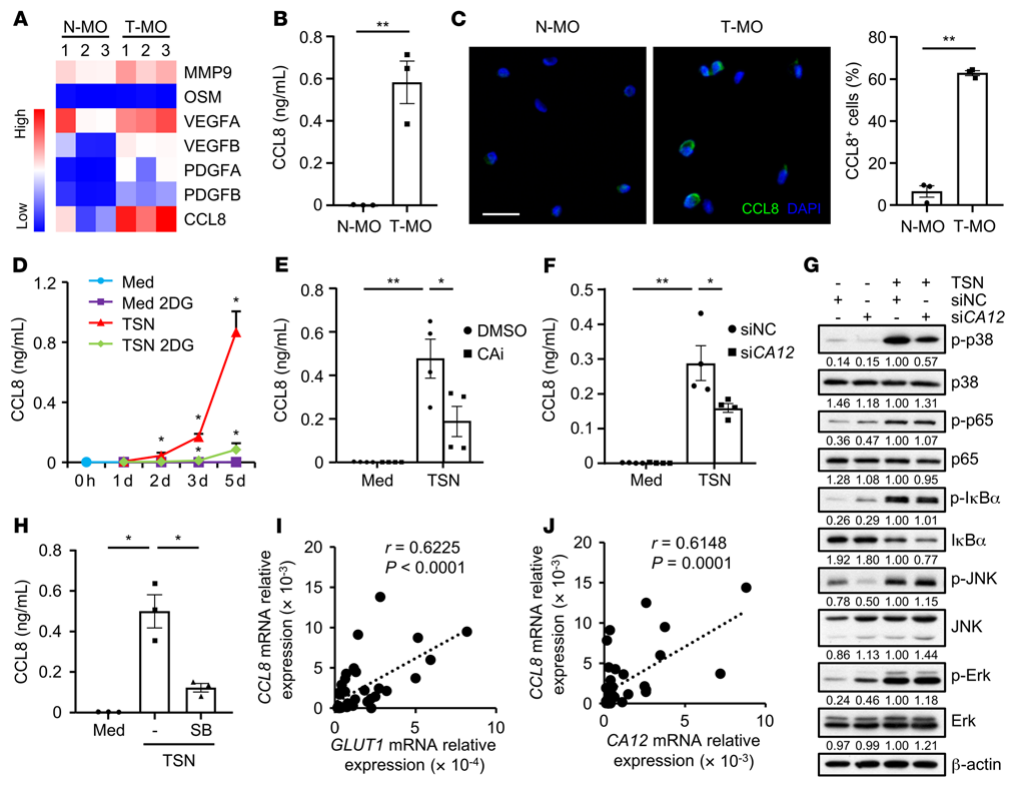
CA12 induces CCL8 production in tumor-associated monocytes and macrophages. (**A**–**C**) CD14^+^ cells were purified from paired nontumor tissues (N-MO) and tumor tissues (T-MO) from 3 patients with HCC. Expression levels of metastasis-related genes were quantified by qPCR (**A**). Levels of CCL8 production by these cells were determined by ELISA (**B**) and confocal microscopy (**C**) (CCL8: green; DAPI: blue). Scale bar: 20 μm. (**D**–**H**) CD14^+^ cells were purified from the peripheral blood of healthy donors. (**D**) Cells were left untreated or treated with HepG2 TSN in the presence or absence of 2DG for the indicated times, and CCL8 production was measured by ELISA (*n* = 3). (**E**) Cells were left untreated or treated with HepG2 TSN in the presence or absence of CAi (100 μM) for 72 hours, and CCL8 production was measured by ELISA (*n* = 4). (**F** and **G**) Cells were transfected with siNC or si*CA12* and then left untreated or treated with HepG2 TSN for 72 hours. Levels of CCL8 production were measured by ELISA (**F**) (*n* = 4). Expression levels of p-p38, p38, p-p65, p65, p-IκBα, IκBα, p-JNK, JNK, p-Erk, and Erk were determined by immunoblotting (**G**) (*n* = 5). (**H**) Cells were left untreated or treated with HepG2 TSN in the presence or absence of SB202190 (SB) (25 μM) for 72 hours, and CCL8 production was measured by ELISA (*n* = 3). (**I** and **J**) CD14^+^ cells were purified from tumor tissues from 36 patients with HCC. Correlations between the mRNA levels of *CCL8* and *GLUT1* or *CA12* in the cells were analyzed by qPCR. Results shown in **B**–**F** and **H** are represented as mean ± SEM. *P* values were obtained by 2-tailed Student’s *t* test (**B** and **C**), 2-way ANOVA (**D**–**F** and **H**), or Pearson’s correlation and linear regression analysis (**I** and **J**). *P < 0.05; **P < 0.01.

By comparing metastasis-related genes expressed in paired nontumor tissue– and tumor tissue–purified monocytes, we found that levels of *MMP9*, *VEGFA*, and *CCL8* were all increased in the tumor monocytes compared with the nontumor monocytes, with *CCL8* exhibiting the most marked upregulation (Figure 5A). The upregulation of CCL8 in tumor-derived monocytes was further confirmed through ELISA and immunofluorescence analysis (Figure 5, B and C).

To explore whether CA12 facilitates tumor metastasis by regulating the production of CCL8 in monocytes, we treated CD14^+^ cells from healthy donors with HepG2 TSN in the presence or absence of the glycolysis inhibitor 2DG, CA12 inhibitor, or si*CA12*. As shown in Figure 5, D–F, 2DG effectively attenuated TSN-induced CCL8 upregulation in monocytes in a time-dependent manner, and the CA12 inhibitor and si*CA12*, compared with their respective controls, also significantly abrogated TSN-induced CCL8 production in these cells. Interestingly, when analyzing signaling pathways that might be influenced by CA12 upregulation in monocytes, we found that, among those observed, including the NF-κB, p38, Erk, and JNK pathways, the increased phosphorylated p38 (p-p38) levels in TSN-exposed monocytes were substantially attenuated by treating these cells with si*CA12*, indicating that the sustained activation of the p38-signaling pathway might be dependent on the upregulation of CA12 (Figure 5G). Moreover, similarly to the CA12 inhibitor and si*CA12*, the p38 inhibitor SB202190 markedly abrogated the increase in CCL8 production in tumor-exposed monocytes (Figure 5H). In support of these in vitro findings, the mRNA levels of *CCL8* showed positive correlations with both *GLUT1* and *CA12* mRNA levels in HCC tumor tissue–purified monocytes (Figure 5, I and J). Together, these data suggest that glycolysis-induced CA12 upregulation might induce CCL8 production by tumor-associated monocytes and macrophages by sustaining the activation of the p38-signaling pathway.

### CCL8 released by tumor-associated monocytes and macrophages induces epithelial-mesenchymal transition and migration of hepatoma cells.

**Figure 6:**
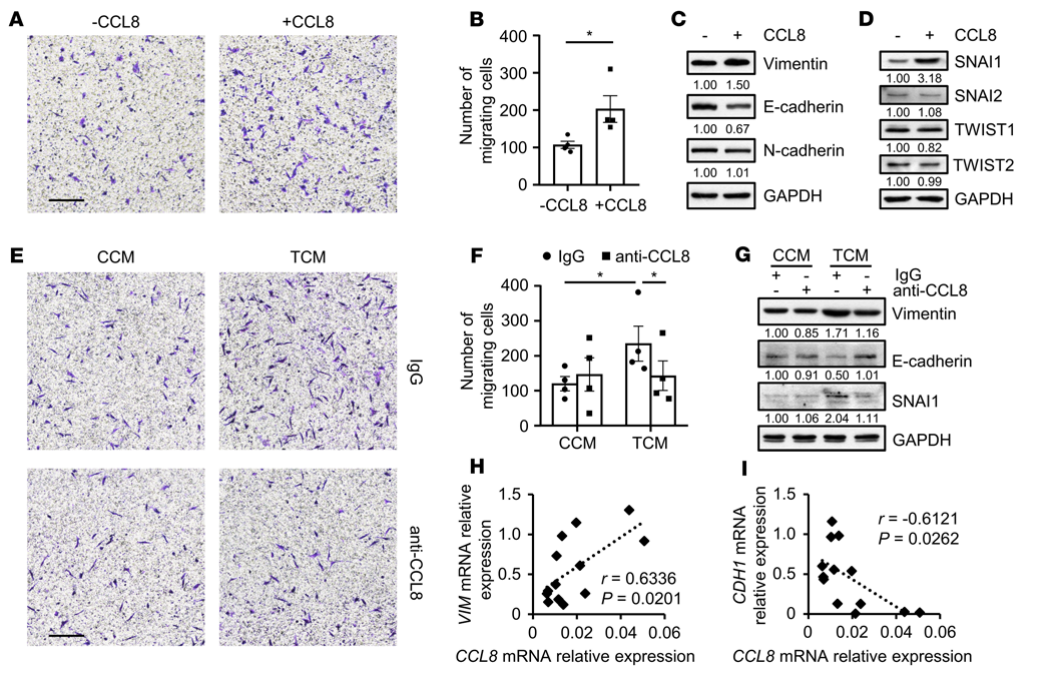
CCL8 released by tumor-associated monocytes and macrophages induces EMT and migration of hepatoma cells. (**A**–**D**) HepG2 cells were left untreated or treated with CCL8 (20 ng/ml) for 20 hours. Cell migration was analyzed (**A** and **B**) (*n* = 4). Expression levels of vimentin, E-cadherin, N-cadherin, SNAI1, SNAI2, TWIST1, and TWIST2 in the cells were determined by immunoblotting (**C** and **D**) (*n* = 5). One out of five representative graphs is shown in **A**. Scale bar: 200 μm. (**E**–**G**) CD14^+^ cells were purified from the peripheral blood of healthy donors. Cells were left untreated or treated with HepG2 TSN for 2 hours, washed, and cultured for another 48 hours before their supernatants were collected. HepG2 cells were then treated with CCM or TCM in the presence or absence of control IgG or anti-CCL8 neutralizing antibody (2 μg/ml) for 20 hours. HepG2 cell migration was analyzed (**E** and **F**) (*n* = 4), and the expression of vimentin, E-cadherin, and SNAI1 was determined by immunoblotting (**G**) (*n* = 5). One out of five representative graphs is shown in **E**. Scale bar: 200 μm. (**H** and **I**) CD14^+^ cells and cancer cells were purified from tumor tissues from 13 patients with HCC. Expression levels of *CCL8* in CD14^+^ cells and *VIM* and *CDH1* expression in cancer cells were determined by qPCR. Correlations between the mRNA levels of *CCL8* and those of *VIM* or *CDH1* were analyzed. Results shown in **B** and **F** are represented as mean ± SEM. *P* values were obtained by 2-tailed Student’s *t* test (**B**), 2-way ANOVA (**F**), or Pearson’s correlation and linear regression analysis (**H** and **I**). *P < 0.05.

In light of the above results, we investigated the hypothesis that CA12 in tumor-associated monocytes might facilitate tumor metastasis by regulating the production of CCL8. As shown in Figure 6, A–D, compared with control treatment, treatment with CCL8 triggered the migration of HepG2 hepatoma cells, substantially increased their levels of vimentin and SNAI1 expression, and downregulated their levels of E-cadherin expression. Notably, supernatants from TSN-exposed healthy donor–derived CD14^+^ cells (conditioned medium from TSN-exposed monocytes [TCM]) could also markedly increase the migration of HepG2 cells in comparison with those from untreated normal monocytes (conditioned medium from control [untreated] monocytes [CCM]), and this increase could be effectively attenuated by treating TCM-exposed cancer cells with anti-CCL8 antibody (Figure 6, E and F). Consistently, the anti-CCL8 antibody abrogated the TCM-induced upregulation of vimentin and SNAI1 expression and attenuated the TCM-induced downregulation of E-cadherin expression in monocytes (Figure 6G). Supporting these in vitro findings, the mRNA levels of *CCL8* in tumor-infiltrating monocytes were found to have some positive correlation with those of *VIM* and some negative association with those of *CDH1* in cancer cells derived from patients with HCC (Figure 6, H and I), and the mRNA levels of *CCL8* in tumor-infiltrating monocytes were also positively correlated with the metastatic potential of HCC patients (*P* = 0.002; Supplemental Table 3).

### Blockade of CA12 reduces macrophage infiltration and induces tumor regression in mice in vivo.

**Figure 7:**
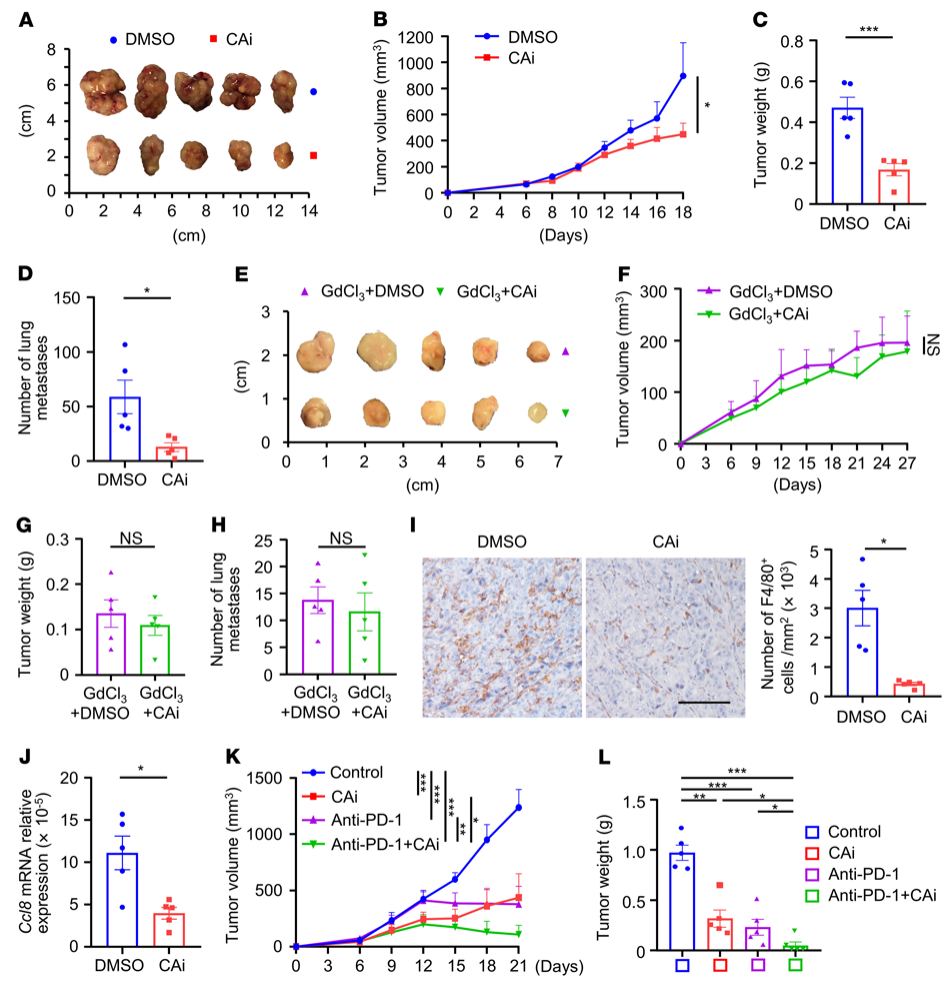
Blockade of CA12 reduces macrophage infiltration and induces tumor regression in mice in vivo. (**A**–**C**) C57BL/6J mice were s.c. injected with Hepa1-6 cells, DMSO or CAi was then i.p. administered. Tumors were excised and photographed (**A**), tumor volumes (**B**) and weights (**C**) were measured. (**D**) An orthotopic tumor model was established by intrahepatic injection of Hepa1-6 cells, DMSO or CAi was then i.p. administered. Lungs were excised from mice, and tumor lung metastases were counted. (**E**–**G**) Mice bearing with subcutaneous tumors were i.p. injected with GdCl_3_ and DMSO or Cai. Tumors were excised and photographed (**E**), tumor volumes (**F**) and weights (**G**) were measured. (**H**–**J**) Mice bearing orthotopic Hepa1-6 tumors were i.p. injected with (**H**) or without (**I** and **J**) GdCl_3_, and treated with DMSO or CAi. Lungs were excised from mice, and tumor lung metastases were counted (**H**). Paraffin-embedded sections of orthotopic hepatic tumors were stained with anti-mouse F4/80 antibody, and infiltration of F4/80^+^ cells was analyzed, scale bar: 500 μm (**I**). CD11b^+^Ly6G^–^ cells were isolated from orthotopic hepatic tumors by FACS, and *Ccl8* expression in these cells were quantified by qPCR (**J**). (**K** and **L**) Mice bearing with subcutaneous tumors were i.p. injected with DMSO together with control IgG (designated as control), CAi, anti-mouse PD-1 antibody, or CAi in combination with the anti-mouse PD-1 antibody. Tumor volumes (**K**) and weights (**L**) were measured. There were 5 representatives for each group in **A**–**L**. Results shown in **B**–**D** and **F**–**L** are represented as mean ± SEM. *P* values were obtained by 2-way ANOVA with Tukey’s correction *t* test (**B**, **F**, and **K**), 2-tailed Student’s *t* test (**C**, **D**, and **G**–**J**), or 1-way ANOVA (**L**). *P < 0.05; **P < 0.01; ***P < 0.001.

Given the selective expression of CA12 on tumor-associated monocytes and macrophages and its important roles in regulating the survival and prometastatic function of these cells, we hypothesized that CA12 might represent an exploitable target for the treatment of HCC. To test this hypothesis, we first examined the effects of a CA12 inhibitor on the number and migration of hepatoma cells themselves. As shown in Supplemental Figure 5, A–D, compared with control treatments, the CA12 inhibitor did not influence the survival or migratory ability of Hepa1-6 cells in vitro, whether under normal culture conditions or lactic acid–induced acidic culture conditions. However, treatment with the CA12 inhibitor did significantly reduce the volumes, weights, and lung metastasis of Hepa1-6 tumors in mice in vivo, compared with the respective control treatments in WT mice (Figure 7, A–D). These in vivo inhibitory effects of the CA12 inhibitor on the growth and metastasis of Hepa1-6 tumors were not observed in mice depleted of macrophages by treatment with GdCl_3_, clodronate liposome, or α-CSF1R (Figure 7, E–H, and Supplemental Figure 6A), indicating that the effects of the CA12 inhibitor were dependent on the availability of monocytes and macrophages. Consistently, while tumor-associated monocytes (human peripheral blood–derived monocytes transfected with siNC and then treated with HepG2 TSN) could increase tumor volume, lung metastasis, and levels of CD14^+^ cell infiltration of HepG2 tumors compared with respective controls in NOD/SCID mice in vivo, no such induction effects were observed in mice treated with CA12-depleted tumor-associated monocytes (monocytes transfected with si*CA12* before being exposed to TSN) (Supplemental Figure 6B). Both immunohistochemical staining and flow cytometry analysis showed that the levels of F4/80^+^ macrophage infiltration in tumor tissues were significantly reduced in CA12 inhibitor–treated WT mice in comparison with untreated control mice (Figure 7I and Supplemental Figure 7A), and the levels of *Ccl8* mRNA expression were significantly reduced in tumor-purified macrophages (Figure 7J). Moreover, the mRNA levels of vimentin expression in tumor tissues were reduced, while those of E-cadherin were increased in CA12 inhibitor–treated WT mice compared with control (Supplemental Figure 7B).

We then explored whether CA12 might represent a myeloid marker whose inhibition could synergistically act with or enhance the effects of lymphoid cell–targeted immune-checkpoint blockade therapeutics in the treatment of HCC. To that end, WT mice s.c. inoculated with Hepa1-6 cells were treated with DMSO, control IgG, CA12 inhibitor, anti–PD-1 antibodies (programmed cell death protein 1 [PD-1]), or the CA12 inhibitor in combination with anti–PD-1 antibodies. As shown in Figure 7, K and L, while the CA12 inhibitor or anti–PD-1 antibodies alone could reduce tumor volumes and weights compared with control treatments, the combination of CA12 and PD-1 blockade exhibited much greater efficacy in terms of attenuating tumor growth in mice in vivo. Moreover, CA12 inhibitor could increase the apoptosis of macrophages and reduce their ratio in total CD45^+^ cells, while anti–PD-1 treatment did not exhibit significant effects on either macrophage apoptosis or their ratio in CD45^+^ cells. While CA12 inhibitor or anti–PD-1 alone could reduce tumor metastasis and enhance mouse survival compared with the control treatment groups, the combination of CA12 and PD-1 blockade exhibited much greater efficacy in terms of attenuating tumor metastasis and enhancing OS of mice in vivo (Supplemental Figure 8, A and B). Notably, neither acetazolamide, a CA12 inhibitor (CAi), nor anti–PD-1 treatment could affect the expression of CD204 and CD206 on tumor-infiltrating macrophages compared with control (Supplemental Figure 8C). As for other cell components, both CA12 inhibitor and anti–PD-1 could increase the ratio of CD8^+^ T cells in total tumor leukocytes, with the combination treatment group exhibiting the most significant induction efficacy (Supplemental Figure 8D). CAi and anti–PD-1 treatment did not significantly affect tumor infiltration ratios of myeloid-derived suppressor cells (MDSCs) and B lymphocytes in total CD45^+^ cells (Supplemental Figure 8D).

Together, these results suggest that targeting CA12 could inhibit tumor progression, presumably through regulation of the accumulation and functions of macrophages, and that CA12 inhibition also exhibited synergistic antitumor effects with immune-checkpoint blockade therapeutics in HCC.

## Discussion

The physicochemical composition of the tumor microenvironment is different from that of the typical interstitium of normal tissue (31, 32). The characteristics of the tumor microenvironment include acidosis, hypoxia, increased lactate levels, reduced glucose concentrations, and secretome changes, which all contribute to an adverse environment that is unfavorable for the survival and function of recruited immune cells (33–36). However, the abundant macrophage population infiltrating HCC tumor tissues indicates that these cells might adopt unique mechanisms to adapt to tumor-specific environments. Our current study identified CA12 as an important regulator mediating the survival and prometastatic function of macrophages in human HCC. The transient metabolic switch in monocytes and macrophages in the peritumoral stroma induced sustained expression of CA12 on these cells through HIF1α- and autocrine cytokine-dependent pathways, and importantly, targeting CA12 could selectively regulate the accumulation and function of macrophages in tumor tissue and induce tumor regression in a mouse model alone or in synergistic combination with immune-checkpoint blockade.

CA9 and CA12, especially the former, have been reported to be tumor associated and to buffer the intracellular pH of cancer cells, thus promoting tumorigenesis and tumor progression (18–20). Inhibition of tumor-associated CA9 is useful in the management of hypoxic tumors that do not respond to classic chemotherapy or radiotherapy, and many types of highly effective CA9 inhibitors have been developed and evaluated in vitro (16). In contrast, the possible role of the CA family in modulating immune cell functions has rarely been investigated. The present study found that CA12 was significantly upregulated in human HCC and that, instead of being expressed by cancer cells, this molecule was primarily concentrated on tumor-infiltrating macrophages in the majority of examined patients and played important roles in mediating the accumulation of these cells. CA12 colocalized with CD206 and CD204, indicating that CA12 might be particularly important for the survival and function of M2-like macrophage subsets in HCC tumor tissues.

Beyond cancer cells, recent studies have reported that cellular metabolic switches were involved in the regulation of immune cells under many physiological and pathological conditions (31, 37–39). However, little is known about whether and how metabolic changes might occur and regulate the phenotypes and functions of immune infiltrates in specific human HCC tumor microenvironments. Our previous studies found that glycolytic activation of peritumoral monocytes, induced by tumor-derived hyaluronan, could increase their expression of PD-L1 via autocrine TNF-α and IL-10 (25). In addition, the present study demonstrates that the transient glycolytic switch in monocytes also induces the stabilization of HIF1α, which acts synergistically with autocrine cytokines to upregulate the sustained expression of CA12 on tumor-associated macrophages. As a canonical oxygen sensor, HIF1α might also be interpreted as an acid sensor for macrophages in this scenario, given its role in mediating the survival of these cells under acidic but not physiologically normal conditions through the regulation of CA12.

Human HCC tumors evolve through long-term crosstalk between cancer cells and their surrounding stroma and can be divided into different areas, such as the peritumoral region, invading edge, and intratumoral region, with each having a distinct structure and composition (40–42). While the peritumoral stroma was enriched with periphery-recruited monocytes, the intratumoral area was primarily infiltrated by phenotypically more matured macrophages (25, 43). In the current study, we treated healthy peripheral blood–derived monocytes in vitro with hepatoma supernatants to induce their differentiation into macrophages with phenotypes similar to those of macrophages in HCC tumor tissues in situ. Our results show that in vitro temporal glycolytic activation in monocytes could lead to sustained expression of CA12 when these cells differentiate into macrophages. Consistently, in situ immunofluorescence staining of human HCC samples showed that, while peritumoral monocytes exhibited enhanced GLUT1 expression compared with those from nontumor or intratumoral areas, both peritumoral and intratumoral infiltrating monocytes and macrophages showed significantly upregulated expression of CA12 compared with those from paired nontumor tissues. The levels of GLUT1 and CA12 expression were positively correlated both in in vitro tumor-exposed monocytes and in their HCC tumor–purified counterparts. These results indicate that, rather than being induced by microenvironments where CA12 is highly expressed, the upregulation of CA12 on macrophages was triggered even before the cells entered the intratumoral regions, a reasonable scenario since it might be better for immune cells to put on “armor” before, not after, stepping onto the battlefield, whether they ultimately act mostly as gatekeepers or enablers in tumors. It was also plausible that the transient metabolic switch in monocytes occurred in the peritumoral areas because the essential component enabling glycolysis, namely, glucose, might be more available in these regions than in the intratumoral region of HCC.

The current data show that CA12 not only mediated the survival of macrophages in lactic acid–conditioned culture medium with a lower pH value than normal control medium, but also directly induced the production of CCL8, which facilitated epithelial-mesenchymal transition (EMT) in cancer cells (44) by tumor-associated macrophages. Accordingly, the levels of CCL8 expression were found to be positively correlated with those of CA12 in tumor-derived monocytes and macrophages and with the metastatic potential of HCC cells. While CCL8 has been previously found to be produced by stromal fibroblasts stimulated with inflammatory cytokines (45–47), the present study showed that, in human HCC, CCL8 was mainly derived from tumor-infiltrating monocytes and macrophages and could be regulated by CA12 expression by these cells. This finding was also consistent with a study showing that CCL8 could be induced in macrophages by TNF-α in breast tumors (44). It has been reported that lactic acid can stimulate the M2 polarization of macrophages in tumor tissues and induce their production of factors such as IL-10 and VEGF (48). Our current study might complement such previous findings by unveiling mechanisms regulating the survival and prometastatic functions of macrophages, thus confirming that multiple factors, not a single factor, are involved in determining the final phenotype and functions of this important immune cell population in tumor tissues. Nonetheless, the selective expression of CA12 on tumor-infiltrating macrophages suggests that CA12 might represent a satisfactory marker for diagnosis and a target for a therapeutic strategy aimed at specifically targeting these cells. Supporting this hypothesis, our data show that the upregulation of CA12 on tumor-associated macrophages could predict increased tumor metastatic potential and reduced survival of patients with HCC and that treatment with a CA12 inhibitor could effectively reduce tumor growth and metastasis in mice in vivo, presumably through attenuating the infiltration and functions of CA12-expressing macrophages.

Although immune-checkpoint blockade could enhance the antitumor effects of the CA12 inhibitor in the mouse model, the underlying mechanism regulating the synergistic or complementary functions of the two agents warrants further validation and exploration. Moreover, while CA12 might mediate the survival of macrophages under acidic conditions, presumably through buffering the intercellular pH of these cells, the detailed molecular pathways and mechanism involved in mediating the prosurvival and CCL8-inducing effects of CA12 are still not clear and require further examination. Nevertheless, our study demonstrates selective expression and an important function of CA12 in HCC tumor–infiltrating macrophages, thus providing a preclinical rationale for the development of CA12-blocking agents as therapeutics for the treatment of human HCC, especially in cases with high levels of macrophage infiltration.

## Methods

### Human HCC tissues and blood samples.

Liver tissue samples were obtained from 121 untreated patients with pathologically confirmed HCC at the Cancer Center of Sun Yat-sen University and Sun Yat-sen Memorial Hospital between 2007 and 2020. Patients with concurrent autoimmune disease, HIV, or syphilis were excluded. Among these patients, 72 (cohort 1) who had complete follow-up data were used for immunohistochemical analysis and assessments of OS and TR. Another 49 (cohort 2) were used for the isolation of tumor-, peritumor- and nontumor-infiltrating leukocytes. Nontumor sites were defined as areas at least 3 cm away from tumor sites, and the peritumoral region was anatomically defined as an area including the tumor invading edge and its close-connected area. To maintain the viability of live cells from fresh tissue and shorten the lapse between tissue acquisition and sample analysis, distinct tissue areas would be generally defined by the surgeons, and a small piece from each region was dissected and given to us for subsequent analysis in our lab. The clinical characteristics of all patients are summarized in Supplemental Table 4. Blood samples were obtained from 60 healthy donors attending the Guangzhou Blood Center (Guangzhou, China). All samples were anonymously coded. Heparin tubes were used to collect blood samples, and all blood assays were performed using fresh cells.

### Isolation of leukocytes or cancer cells from peripheral blood or tissues.

Human peripheral blood samples from healthy donors were separated by Ficoll density gradient centrifugation at 450*g*. The leukocyte layer was collected to isolate CD14^+^ monocytes using magnetic beads, and the other cells were collected as CD14^–^ leukocytes.

Tumor-, peritumor-, and nontumor-infiltrating leukocytes were obtained from paired fresh tissue samples, as described previously (41). Briefly, fresh HCC biopsy specimens were cut into very small pieces and digested in RPMI 1640 medium supplemented with 0.002% DNase I, 0.05% collagenase IV, 20% FBS, 100 U/ml penicillin, and 100 μg/ml streptomycin at 37°C for 40 minutes. Dissociated cells were separated by Ficoll density gradient centrifugation at 450*g*. CD14^+^ and CD14^–^ leukocytes were isolated as described above.

For isolation of primary cancer cells, HCC tumor biopsy specimens were digested and separated by Ficoll density gradient centrifugation, at 450*g*, as described above. The bottom layer consisting of cancer cells with erythrocytes was washed and collected. The contaminating erythrocytes were removed by using ACK lysis buffer (TBD Science).

For isolation of tumor-infiltrating leukocytes from mouse orthotopic hepatic tumors, fresh tumors were cut into small pieces and digested in RPMI 1640 medium supplemented with 0.002% DNase I, 0.05% collagenase IV, 50 μg/ml hyaluronidase, 30 μg/ml collagenase XI, 20% FBS, 100 U/ml penicillin, and 100 μg/ml streptomycin at 37°C for 40 minutes. Dissociated cells were separated by Ficoll density gradient centrifugationat 450*g*. Leukocytes were stained with PE/cyanine 7–conjugated anti-mouse CD11b, BV421-conjugated anti-mouse Ly6C, FITC-conjugated anti-mouse Gr-1, and PE-CF594–conjugated anti-mouse Ly6G antibodies. CD11b^+^Ly6G^–^ monocytes and macrophages were then purified using a MoFlo XDP Cell Sorter (Beckman Coulter). Cancer cells were collected in ways similar to those used for human HCC primary cancer cells, as described above. The purified cells were then used for direct analysis, in vitro experiments, or collection of culture supernatants.

### Cell lines.

Tumor cell lines (HepG2, SK-Hep-1, Hep3B, PLC/PRF/5, and Hepa1-6) were purchased from ATCC. The normal human liver cell line HL-7702 was purchased from the Institute of Biochemistry and Cell Biology at the Chinese Academy of Science (Shanghai, China). All cell lines were regularly tested for mycoplasma contamination using the single-step PCR method and were maintained in DMEM supplemented with 10% v/v FBS, 100 U/ml penicillin, and 100 μg/ml streptomycin.

### Preparation of culture supernatants from cell lines or monocytes.

Cell line supernatants were prepared by plating 5 × 10^6^ tumor cells in 10 ml of complete DMEM in 100 mm dishes for 24 hours and then changing the medium to complete DMEM supplemented with 10% v/v human AB serum instead of FBS. After 2 days, supernatants were harvested, centrifuged 450*g* for 10 minutes, and then 1500*g* for 15 minutes, and stored in aliquots at –80°C.

CD14^+^ cells were purified from the peripheral blood of healthy donors as described above. These cells were left untreated or treated with HepG2 hepatoma supernatants (TSN) for 2 hours and washed and cultured for another 48 hours before their supernatants (designated CCM and TCM, respectively) were harvested, centrifuged 450*g* for 10 minutes, and then 1500*g* for 15 minutes, and stored at –80°C before use.

### In vitro culture of monocytes.

Purified CD14^+^ cells were cultured in DMEM supplemented with 10% human AB serum in the presence or absence of 30% v/v HepG2, SK-Hep-1, Hep3B, PLC/PRF/5, or HL-7702 TSNs. In some experiments, CD14^+^ cells were left untreated or treated with TSN in the presence or absence of 2DG (25 mM), 3PO (25 μM), DMOG (200 μM), echinomycin (10 nM), CAi (100 μM), SB202190 (25 μM), anti–TNF-α neutralizing antibody (10 μg/ml), anti–IL-6 neutralizing antibody (20 μg/ml), anti–IL-10 neutralizing antibody (10 μg/ml), anti–IL-1β neutralizing antibody (10 μg/ml), human recombinant TNF-α (10 ng/ml), human recombinant IL-6 (10 ng/ml), human recombinant IL-10 (1 ng/ml), or human recombinant IL-1β (1 ng/ml) for the indicated times. In some other experiments, monocytes were pretreated with DMSO or TEPP-46 (100 μM) for 1 hour or transfected with control siRNA or si*CA12* before being exposed to medium or HepG2 TSN.

In other experiments, monocytes were transfected with siNC or si*CA12* and then treated with or without HepG2 TSN for 48 hours before being exposed to pH 7.2 control medium, pH 6.9 HCO3^–^–free DMEM medium, pH 6.3 HCO3^–^-free DMEM medium, or L-lactic acid (20 mM) for the indicated times.

### Quantitative real-time PCR.

Total RNA was extracted using TRIzol reagent and then used to synthesize cDNA with 5X All-In-One RT MasterMix. Sequences of the primers used for PCR analysis were listed in Supplemental Table 5. Quantitative real-time PCR (qPCR) was performed according to a standard protocol using SYBR Green Real-Time PCR Mix in the LightCycler 480 System (Roche). To determine the relative fold change of different genes, their levels of expression were normalized to those of human *ACTB* or mouse *Actb*.

### Immunofluorescence staining.

For immunofluorescence analysis of patient samples, frozen sections were processed as described previously (25). The sections were incubated with anti-human CD68 and anti-human CA12 antibodies; anti-human CD68, anti-human CA12, and anti-human Glut1 antibodies; anti-human CD68, anti-human CA12, and anti-human CD204 antibodies; or anti-human CD68, anti-human CA12, and anti-human CD206 antibodies. Immunofluorescence signals were amplificated by a tyramide signal amplification kit (PANO 7 IHC kit [TSA-RM]; Panovue) as instructed by the manufacturer for visualization.

For immunofluorescence staining of cultured cells, cells growing on coverslips were treated with or without Brefeldin A (5 μg/ml) for 4 hours before being fixed by cold methanol for 15 minutes at room temperature, rinsed with PBS, permeabilized, and blocked by PBS containing 5% BSA and 0.3% Triton X-100 for 1 hour at room temperature. Cells were then incubated with primary antibodies against human CCL8 or CA12, respectively, overnight at 4°C before being exposed to Alex Flour 488–conjugated donkey anti-rabbit IgG. Nuclei were counterstained with DAPI. In other experiments, cultured cells were stained with SYTOX Green for 1 hour before visualization.

Immunofluorescence staining images were visualized using a ZEISS microscope (LSM880), and positive cells were quantified using ImagePro Plus software Version 6.0 (Media Cybernetics) and expressed as mean ± SEM in high-powered fields.

### Immunoblotting.

Immunoblotting was performed as described previously (43). Primary antibodies used included the following: anti-human CA12, p-p38, p38, p-p65, p65, p-IκBα, IκBα, p-JNK, JNK, p-Erk1/2, Erk1/2, vimentin, E-cadherin, N-cadherin, SNAI1, SNAI2, TWIST1, TWIST2, HIF1α, β-actin, and GAPDH. HRP-linked goat anti-rabbit/mouse IgG antibodies were purchased from Cell Signaling Technology.

### Flow cytometry.

Cell-surface markers were determined by direct staining with anti-human CD14-AF700, anti-human PD-L1-PE, anti-human CD80-PE, anti-human CD86-APC, anti-human HLA-DR-BV421, anti-mouse CD45-BV570, anti-mouse F4/80-APC, anti-mouse CD204-PE, anti-mouse CD206-APC, anti-mouse F4/80-PC7, anti-mouse CD8-ef450, anti-mouse B220-BV421, anti-mouse CD11b-AF700, anti-mouse Gr1-FITC, anti-mouse CD3-PE, or anti-mouse Gr1-APC antibodies. To determine the expression of CA12, leukocytes were stained with CA12 antibody before being stained with Alex Flour 488–conjugated donkey anti-rabbit IgG. In some experiments, monocytes or Hepa1-6 cells were stained with annexin V and PI following instructions for the Annexin V Apoptosis Detection Kit (Thermo Fisher Scientific). Data were acquired with the CytoFLEXS flow cytometer (Beckman Coulter) and evaluated with FlowJo software, version 10 (Tree Star).

### Immunohistochemistry staining.

Paraffin-embedded and formalin-fixed samples were cut into 5 μm sections; this was followed by procedures for immunohistochemistry, as described previously (43). After incubation with primary antibodies against human CD68 and CA12, mouse F4/80, or human CD14, sections were stained with the corresponding secondary antibodies and visualized with diaminobenzidine and 3-amino-9-ethylcarbazole in an EnVision System (Dako).

### Evaluation of immunohistochemical variables.

Analysis was performed by 2 independent observers who were blinded to the clinical outcome. At low-power field (×100), tissue sections were screened, and the 5 most representative fields were selected using an Eclipse Ni-U highly versatile upright microscope combining system (Nikon Instruments). For evaluating the density of tissue-infiltrating CD68^+^ macrophages (49–51), CD68^+^CA12^+^ cells, F4/80^+^ cells, or CD14^+^ cells, the respective areas of tissues were then scanned at ×400 magnification (0.146 mm^2^ per field). The number of nucleated cells was then counted manually and expressed as cells per field or cells per mm^2^.

### Lactate production assay.

Concentration of lactate in mediums was measured by L-Lactate Assay Kit according to the manufacturer’s instruction (Eton Bioscience).

### RNA interference.

Purified monocytes were left untreated or transfected with 300 nM control siRNA, si*CA12*, or si*HIF1A* using The P3 Primary Cell 4D-Nucleofector X Kit with Lonza 4D Nucleofector (Lonza). All siRNA duplexes were purchased from GenePharma, and their sequences are listed in Supplemental Table 5.

### ELISA.

Cytokine concentrations were detected by ELISA kits according to the manufacturer’s instructions: TNF-α, 88-7346-86, eBioscience; IL-6, 88-7066-88, eBioscience; IL-10, 88-7106-88, eBioscience; IL-1β, 88-7261-88, eBioscience; CCL8, 442204, BioLegend).

### Cell-migration assay.

HepG2 cells were left untreated or treated with CCL8 (20 ng/ml), CCL8-neutralizing antibody (2 μg/ml), or IgG1 isotype control in the presence or absence of CCM or TCM for 20 hours. Hepa1-6 cells were treated with medium or L-lactic acid (20 mM) in the presence or absence of DMSO or CAi (100 μM) for 24 hours. The migration assays for differently treated HepG2 or Hepa1-6 cells were then performed using a 24-well Boyden chamber with an 8 μm polycarbonate membrane. Briefly, cancer cells (3 × 10^4^) in 100 μl of serum-free DMEM were added to the upper compartment of the chamber, while the lower compartment was filled with 600 μl DMEM containing 10% FBS. After 10 hours of incubation, cells remaining on the upper surface of the membrane were removed. The migrated tumor cells on the lower surface of the membrane were rinsed with PBS and subsequently stained with crystal violet after fixation and counted under a light microscope (Nikon Instruments).

### Animals.

WT male C57BL/6J mice were purchased from the Guangdong Medical Laboratory Animal Center, and male NOD/ShiLtJGpt-*Prkdc^em26Cd52^*/Gpt mice (referred as to NOD/SCID) were purchased from GemPharmatech. All mice were maintained under specific pathogen–free conditions and were used at between 6 and 8 weeks of age.

### Mouse tumor models and treatments.

For the s.c. tumor model, a total of 8 × 10^5^ Hepa1-6 cells were subcutaneously transplanted into the flanks of mice. Tumor volumes were measured along 2 orthogonal axes (a and b), and tumor volumes were calculated (a^2^ × b/2). Tumors were grown for 6 days to reach a size of 80 mm^3^. For the orthotopic hepatic tumor model, a total of 4 × 10^5^ Hepa1-6 cells were suspended in 25 μl of 66.7% basement membrane extract and intrahepatically injected into the left lobe of the liver of anesthetized 6-week-old C57BL/6J mice. In some experiments, 5 × 10^5^ HepG2 cells were used to establish orthotopic hepatic tumor models in NOD/SCID mice. For tumor growth, an s.c. model was applied so that the tumor volumes at indicated time points could be measured without sacrificing the mice. For lung metastasis experiments, an orthotopic model was applied, since it was supposed to better mimic the metastatic process of human liver cancer.

In some experiments, DMSO or 20 mg/kg CAi was administered i.p. every day beginning on day 6. In some experiments, GdCl_3_ (10 mg/kg, every 3 days beginning on day 0), control liposome/clodronate liposome (52.5 mg/kg were injected at 24 hours before the orthotopic transplantation of Hepa1-6, and 35 mg/kg twice a week thereafter), or control IgG/α-CSF1R (10 mg/kg, every 3 days beginning on day 2) was administered i.p., with DMSO or CAi injected i.p. every day beginning on day 6. In some experiments, 10^6^ monocytes purified from the peripheral blood of healthy donors were left untreated, transfected with siNC, and then treated with HepG2 TSN or transfected with si*CA12* before being exposed to TSN for 48 hours. These cells were then i.p. injected into NOD/SCID mice bearing orthotopic HepG2 tumors twice a week. In other experiments, DMSO together with IgG2a isotype control antibody (designated as control), CAi, 25 μg of anti-mouse PD-1 antibody, or CAi in combination with the anti-mouse PD-1 antibody were i.p. injected beginning on day 6 (DMSO and CAi, every day; antibodies, every 3 days).

The tumor volumes of different treatment groups were measured every 2 or 3 days. At the indicated times, tumors were excised, and their weights were analyzed. To confirm the presence of lung metastases, paraffin-embedded and formalin-fixed lungs were cut into 7 μm sections and then stained with H&E. The survival of mice from different treatment groups was measured at humane end points, as previously described (52), and the expression of different markers in tumor tissues was determined by immunohistochemistry staining or flow cytometry, as described above.

### Statistics.

Statistical tests used are indicated in the figure legends. The results are expressed as mean ± SEM. Correlations between parameters were measured by Pearson’s correlation. Statistical analysis was performed with GraphPad Prism 6. Survival curves were calculated by the Kaplan-Meier method and analyzed by the log-rank test. Cox’s proportional hazards model was used to identify prognostic factors through univariate and multivariate analyses. *P* values were assessed using 2-tailed Student’s *t* test or 1-way or 2-way ANOVA with the following thresholds for statistical significance: *P* < 0.05; *P* < 0.01; and *P* < 0.001.

### Study approval.

For experiments using human samples, all samples were anonymously coded in accordance with local ethical guidelines (as stipulated by the Declaration of Helsinki). Written, informed consent was obtained from all patients, and the study protocol was approved by the IRB of the Sun Yat-sen University Cancer Center (Guangzhou, China). All animal experiments were performed with the approval of the IACUC of the Sun Yat-sen University (Guangzhou, China).

For further details regarding the antibodies and reagents used in this study, please refer to Supplemental Table 6 and Supplemental Table 7.

## Author contributions

WRN designed the experiments, processed tissues, performed flow cytometry, immunoblotting, and mouse studies, collected data, and wrote the paper. DJ and YFH performed immunohistochemical and immunofluorescence staining and analyzed the data. XCL helped with and performed real-time PCR and lactate production detection. ZPP and ZZJ helped with and performed ELISA and cell migration assay TK, SMZ, YW, and LZ advised and consulted on analyzing human data and/or supported the project. YW and LZ planned and supported the project, analyzed data, and wrote the paper.

## Supplementary Material

Supplemental data

## Version 1 04/01/2022

Electronic publication
