# Supplementary material for: Carbonic anhydrase XII mediates the survival and prometastatic functions of macrophages in human hepatocellular carcinoma
Source: J Clin Invest. 2022 Apr 1;132(7):e153110. doi: 10.1172/JCI153110 (PMC8970669; doi:10.1172/JCI153110)
Supplement: Supplemental data [file jci-132-153110-s128.pdf]

Supplementary Materials for

Carbonic anhydrase XII mediates the survival and pro-metastatic functions of macrophages in human hepatocellular carcinoma

Wan-Ru Ning, Da Jiang, Xing-Chen Liu, Yu-Fan Huang, Zhi-Peng Peng, Ze-Zhou Jiang, Tiebang Kang, Shi-Mei Zhuang, Yan Wu*, Limin Zheng*

*Corresponding authors. Email: zhenglm@mail.sysu.edu.cn (L.Z.); wuyan32@mail.sysu.edu.cn (Y.W.)

This PDF file includes:

Supplemental Figures 1 to 8

Supplemental Tables 1 to 7

A

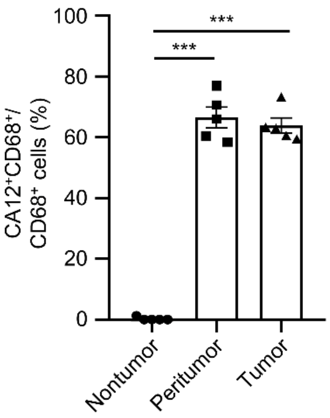

Supplemental Figure 1. CA12 is upregulated on tumor-infiltrating monocytes/macrophages. Frozen sections of HCC samples were stained with anti-human CD68 antibody, anti-human CA12 antibody, and DAPI. The colocalization and distribution of cell signals were analyzed by confocal microscopy. Ratios of $CA12^{+}CD68^{+}$ cells in total $CD68^{+}$ cells in different tissue regions were analyzed. n = 5, one-way analysis of variance (ANOVA) was used for statistical analysis. ***P < 0.001.

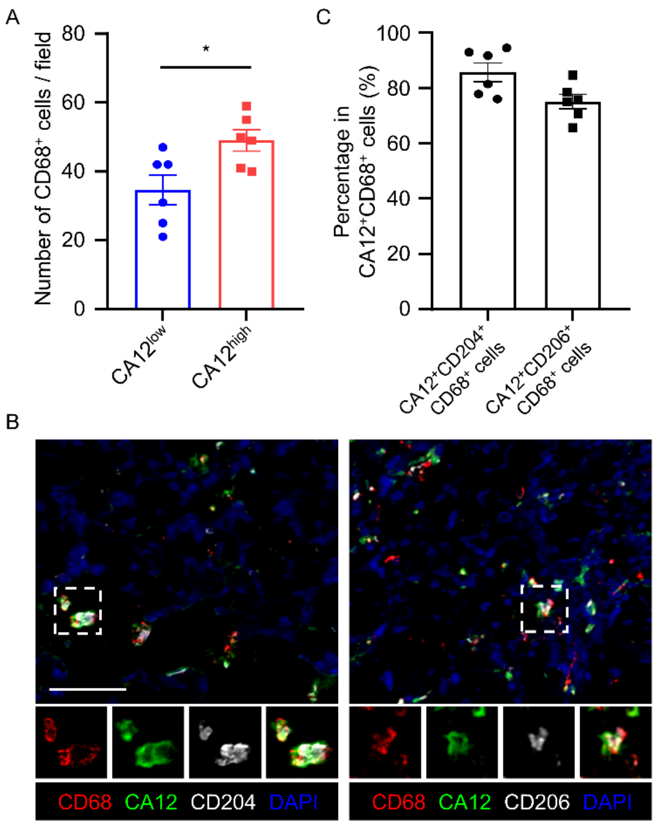

Supplemental Figure 2. Correlation with CD68 and co-localization with CD204 and CD206 signals of CA12 on HCC tumor infiltrating monocytes/macrophages. (**A**) Lymphocytes were purified from tumor tissues of 12 HCC patients. Their levels of *CA12* expression were measured by qPCR. Frozen sections from the same patients were stained with anti-human CD68 antibody, and densities of $CD68^{+}$ cells infiltration were calculated under confocal microscopy. Patients were then divided into 2 groups according to their median value of CA12 expression in tumor tissues. Levels of $CD68^{+}$ cells infiltration between the $CA12^{high}$ and $CA12^{low}$ group was compared. Two-tailed Student's *t*-test was used for statistical analysis. The results were expressed as the mean ± SEM. *$P < 0.05$. (**B** and **C**) Frozen sections of HCC samples were stained with anti-human CD68 antibody, anti-human CA12 antibody, anti-human CD204 antibody, and DAPI; or anti-human CD68 antibody, anti-human CA12 antibody, anti-human CD206 antibody, and DAPI. The

colocalization and distribution of different cell signals were visualized (**B**). Ratios of $CA12^+CD204^+CD68^+$ or $CA12^+CD206^+CD68^+$ cells in total $CA12^+CD68^+$ cells in tumor tissues were analyzed (**C**). Red: CD68, green: CA12, white: CD204/CD206, blue: DAPI. Scale bar: 50 μm. One out of 5 representative micrographs from 6 independent experiments was shown. HCC, hepatocellular carcinoma; CA12, carbonic anhydrase XII; qPCR, quantitative real-time PCR.

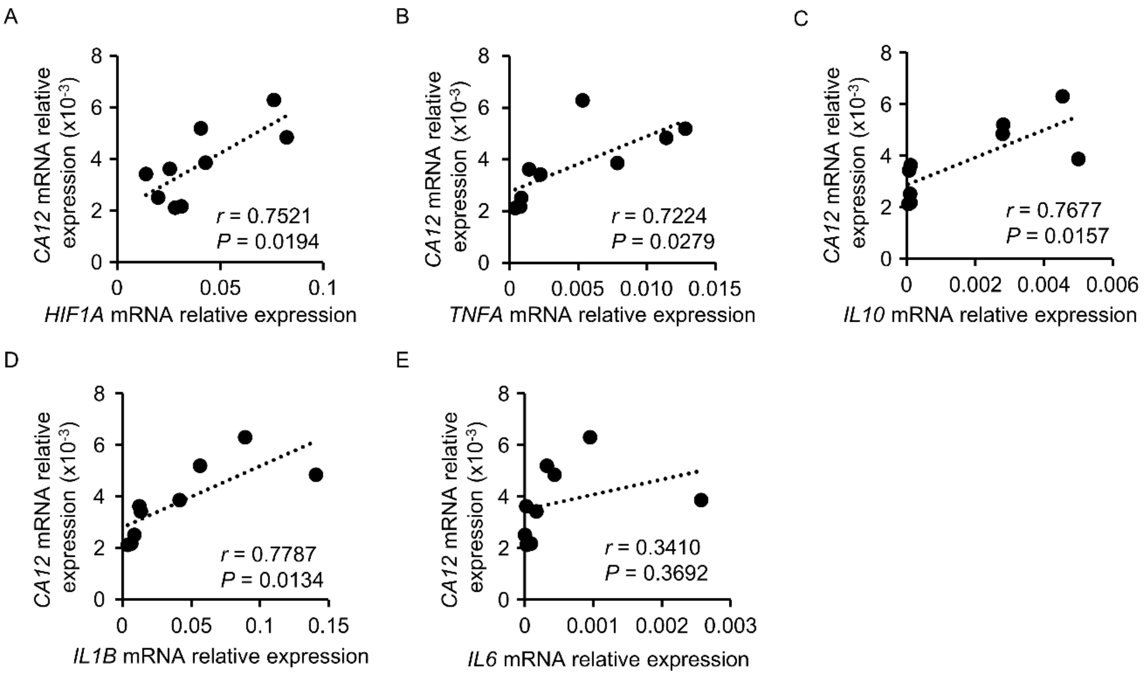

Supplemental Figure 3. The level of CA12 expression is positively correlated with those of HIF1α and autocrine cytokines in HCC tumor-infiltrating monocytes/macrophages. (A-E) Fresh $CD14^+$ cells were purified from tumor tissues from 9 patients with HCC. The expression of *CA12*, *HIF1A* and cytokines was determined by qPCR, and the correlations between the levels of CA12 and HIF1α or cytokines in the cells were analyzed. Pearson correlation and linear regression analysis was used for statistical analysis. HCC, hepatocellular carcinoma; CA12, carbonic anhydrase XII; HIF1A, hypoxia-inducible factor 1 alpha; IL, interleukin; qPCR, quantitative real-time PCR.

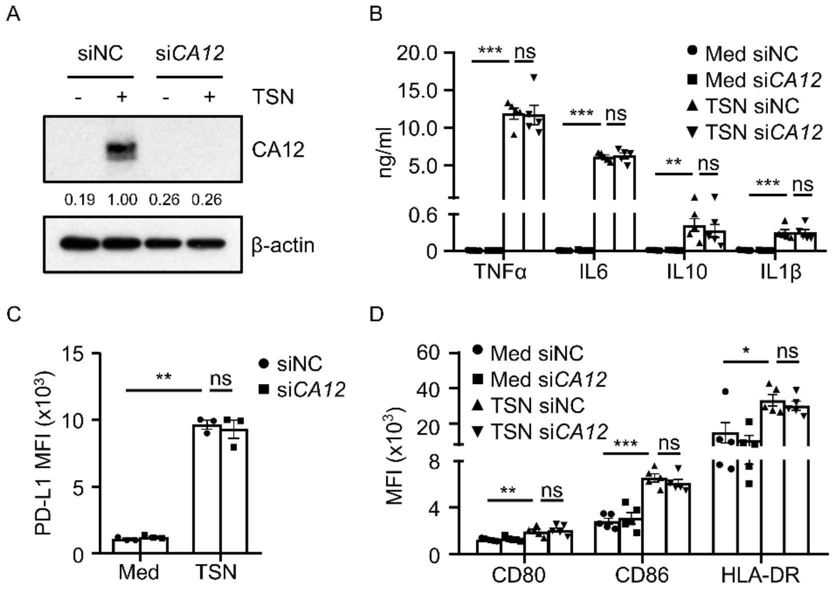

Supplemental Figure 4. si*CA12* did not influence the TSN-induced production of cytokines and expression of some surface markers. $CD14^+$ cells were purified from the peripheral blood of healthy donors. Cells were transfected with siNC or si*CA12* and then treated with or without HepG2 TSN for 48 hours. (**A**) The expression levels of CA12 were determined by western blotting ($n = 5$). (**B**) The levels of TNFα, IL6, IL10 and IL1β production were measured by ELISA ($n = 5$). (**C**) The expression levels of PD-L1 were analyzed by flow cytometry ($n = 3$). (**D**) The expression levels of CD80, CD86, and HLA-DR were analyzed by flow cytometry ($n = 5$). Results shown in **B**, **C** and **D** are expressed as the mean ± SEM. P values were obtained by two-way ANOVA. *$P < 0.05$, **$P < 0.01$, ***$P < 0.001$. CA12, carbonic anhydrase XII; TSN, tumor culture supernatants; TNF, tumor necrosis factor; IL, interleukin; ELISA, enzyme-linked immunosorbent assay; PD-L1, programmed death-ligand 1; ANOVA, analysis of variance; ns, no significance.

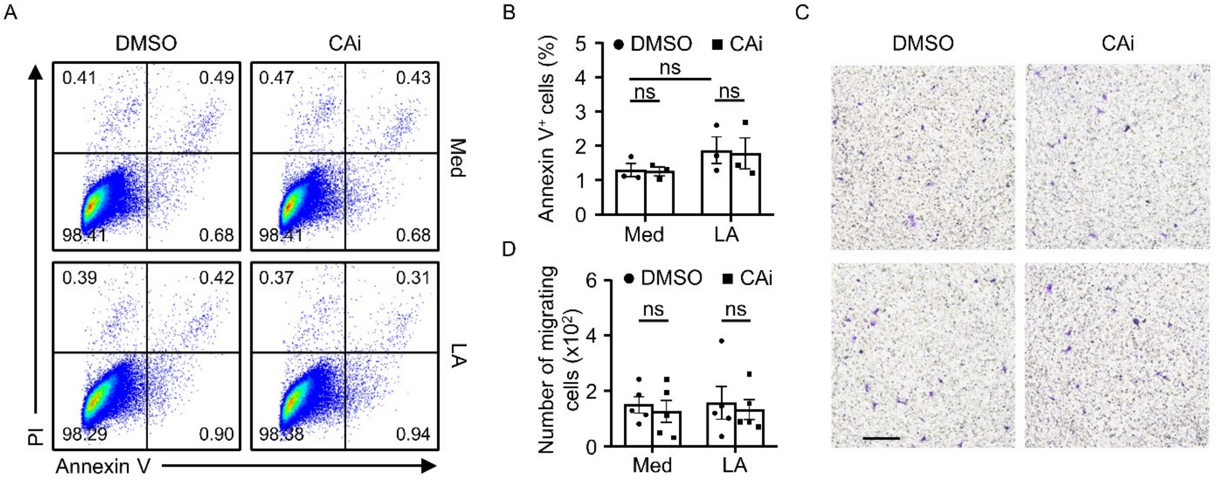

Supplemental Figure 5. CA12 inhibitor does not directly affect the survival and migration of cancer cells. Hepa1-6 cells were treated with medium (Med) or L-lactic acid (LA, 20 mM) in the presence or absence of DMSO or CAi for 24 hours. (**A** and **B**) Apoptosis of these cells was analyzed by flow cytometry (n = 3). (**C** and **D**) Migration of these cells was visualized and calculated (n = 5). Scale bar: 200 µm. Results shown in **B** and **D** are expressed as the mean ± SEM. P values were obtained by two-way ANOVA (**B** and **D**). CA12, carbonic anhydrase XII; DMSO, dimethyl sulfoxide; CAi, CA12 inhibitor; ANOVA, analysis of variance; ns, no significance.

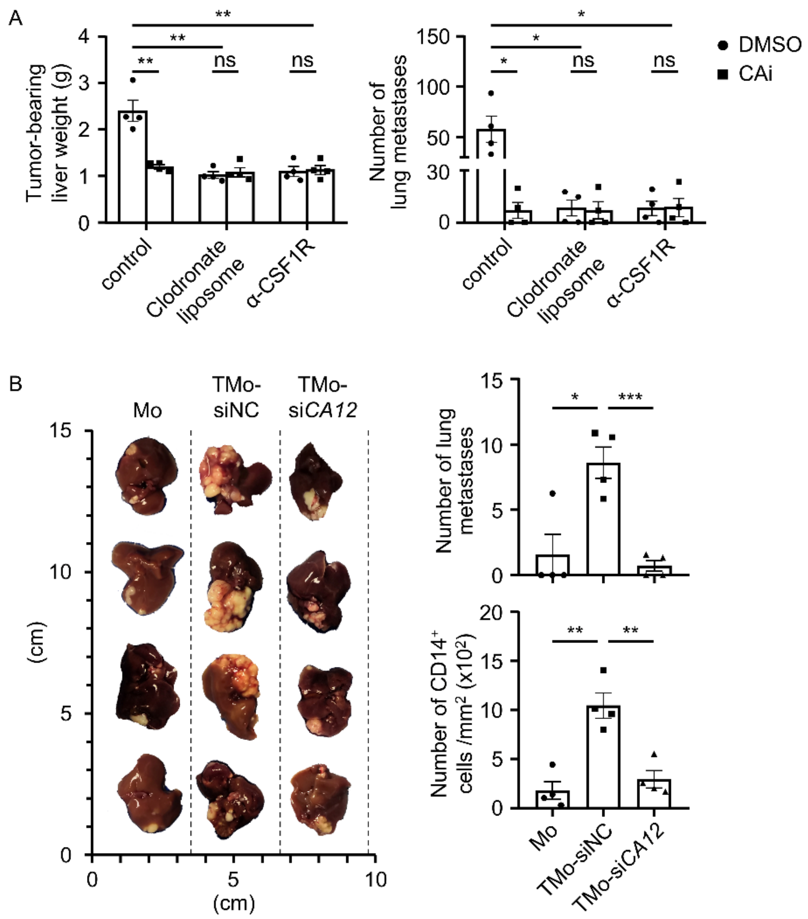

Supplemental Figure 6. Effects of the CA12 inhibitor on tumor-bearing mice are dependent on the availability of monocytes/macrophages. (**A**) Wildtype mice bearing orthotopic Hepa1-6 tumors were i.p. injected with control agents (control liposome (52.5 mg/kg injected 24 hours before the orthotopic transplantation of Hepa1-6, and 35 mg/kg twice a week thereafter) + control IgG (10mg/kg, every 3 days beginning on day 2)), or clodronate liposome (52.5 mg/kg injected 24 hours before the orthotopic transplantation of Hepa1-6, and 35 mg/kg liposome twice a week thereafter), or α-CSF1R (10mg/kg, every 3 days beginning on day 2), AND DMSO or CAi every day beginning on day 6. On day 24, livers and lungs were excised from mice in different treatment groups, and tumor-bearing-liver weights and tumor lung metastases were measured or counted respectively. (**B**) Monocytes purified from the peripheral blood of healthy donors were left

untreated (Mo), transfected with siNC and then treated with HepG2 TSN for 48 hours (TMo-siNC), or transfected with si*CA12* before being exposed to TSN for 48 hours (TMo-si*CA12*). These cells were then i.p. injected into NOD-SCID mice bearing orthotopic HepG2 tumors twice a week. On day 24, tumors and lungs were excised from mice in different treatment groups. Tumor volumes and tumor lung metastases were measured. Paraffin-embedded sections of tumor tissues were stained with anti-human CD14 antibody, and the infiltration of $CD14^+$ cells was analyzed. There were 4 representatives for each group in **A** and **B**. Results are expressed as the mean ± SEM. *P* values were obtained by two-tailed Student's *t*-test (**A**), or two-way ANOVA (**B**). $*P < 0.05$, $**P < 0.01$, $***P < 0.001$. CA12, carbonic anhydrase XII; DMSO, dimethyl sulfoxide; CAi, CA12 inhibitor; CSF1R, colony-stimulating factor 1 receptor; ANOVA, analysis of variance; ns, no significance.

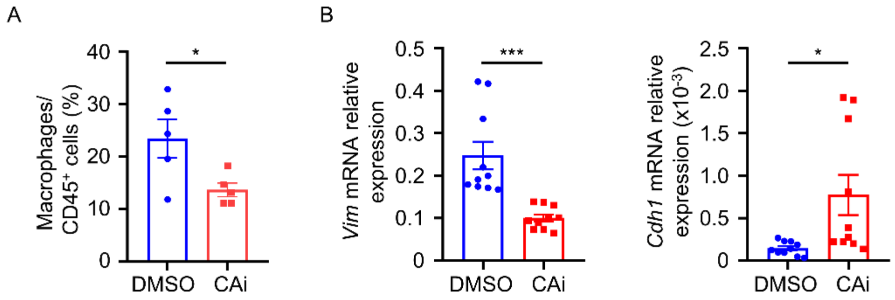

Supplemental Figure 7. CA12 inhibitor reduces levels of F4/80+ macrophages infiltration and impacts EMT-related markers expression in tumor bearing mice. An orthotopic hepatic tumor model was established by subcapsular intrahepatic injection of Hepa1-6 cells on day 0. DMSO or CAi was then intraperitoneally administered every day beginning on day 6. (**A**) Tumors were excised at day 24 and leukocytes were isolated from differently-treated tumors. Levels of F4/80+ macrophages infiltration in tumor tissues were determined by flow cytometry ($n = 5$ for each group). (**B**) Tumors were excised at day 24 and levels of Vimentin (*Vim*) and E-cadherin (*Cdh1*) expression in tumor tissues were measured by qPCR ($n = 10$ for each group). Results are expressed as the mean ± SEM. Two-tailed Student's *t*-test was used for statistical analysis. *$P < 0.05$, ***$P < 0.001$. CA12, carbonic anhydrase XII; DMSO, dimethyl sulfoxide; CAi, CA12 inhibitor; qPCR, quantitative real-time PCR.

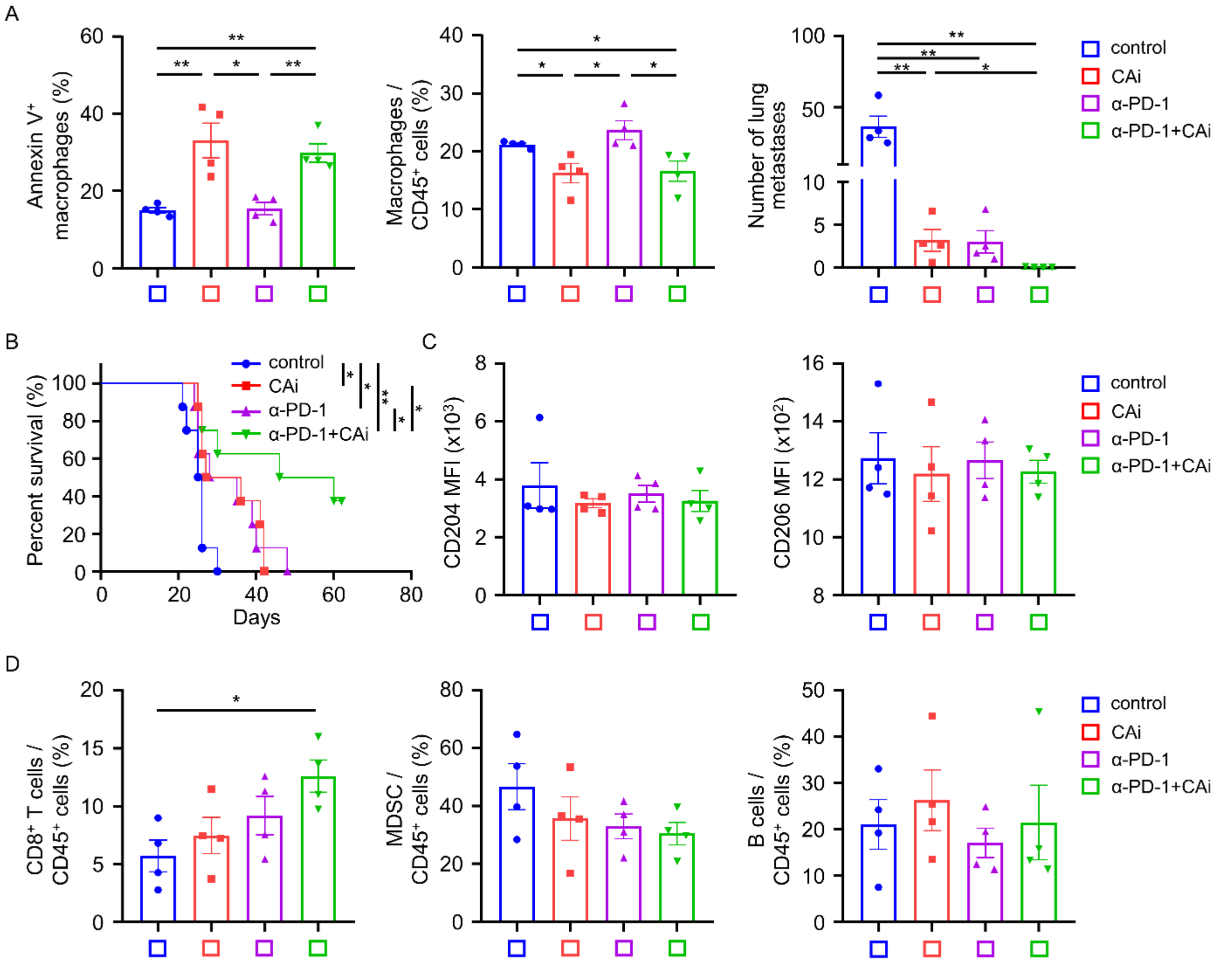

Supplemental Figure 8. Effects of combinational treatment with CA12 inhibitor and anti-PD-1 antibody on tumor-bearing mice. (A-C) C57BL/6J mice with orthotopic Hepa1-6 tumors were treated with DMSO together with control IgG (designated as control), CA12 inhibitor (CAi), anti-mouse PD-1 antibody, or CAi in combination with anti-mouse PD-1 antibody. Lung metastases and mice survival were analyzed, and the expression of different markers in tumor tissues were determined by flow cytometry. There were 4 representatives for each group in **A**, **C** and **D**, and there were 8 representatives for each group in **B**. Results are expressed as the mean ± SEM. *P* values were obtained by two-tailed Student's *t*-test (**A**, **C** and **D**), or the log-rank test (**B**). $^{*}P < 0.05$, $^{**}P < 0.01$. α-, anti-; DMSO, dimethyl sulfoxide; CAi, CA12 inhibitor; PD-1, programmed cell death protein 1; MDSC, myeloid-derived suppressor cell.

Supplemental Table 1. Univariate and multivariate analysis of factors associated with overall survival and tumor recurrence of patients with HCC.

	OS				TR			
	Univariate	Multivariate			Univariate	Multivariate		
Variables	*P* value	HR	95% CI	*P* value	*P* value	HR	95% CI	*P* value
Age, years (>50 vs ≤50)	0.315			n.a.	0.715			n.a.
Gender (male vs female)	0.255			n.a.	0.294			n.a.
HBsAg (positive vs negative)	0.862			n.a.	0.075			n.a.
Cirrhosis (present vs absent)	0.057			n.a.	0.524			n.a.
ALT, U/L (>40 vs ≤40)	0.340			n.a.	0.800			n.a.
AFP, ng/ml (>25 vs ≤25)	0.157			n.a.	0.542			n.a.
Tumor size, cm (>5 vs ≤5)	0.090			n.a.	0.145			n.a.
Tumor multiplicity (multiple vs solitary)	**< 0.001**	4.918	0.871-27.788	0.071	**0.011**	1.463	0.488-4.381	0.497
Vascular invasion (present vs absent)	0.089			n.a.	0.216			n.a.
Metastatic potential [A] (yes vs no)	0.109			n.a.	0.290			n.a.
TNM stage (II + III vs I)	**0.002**	0.727	0.124-4.256	0.724	**0.007**	1.346	0.452-4.009	0.593
Tumor differentiation (III + IV vs I + II)	0.167			n.a.	0.678			n.a.
Intratumoral $CD68^+CA12^+$ cells (high vs low)	**0.008**	2.734	1.099-6.799	**0.030**	**0.005**	2.003	1.087-3.690	**0.026**

Cox proportional hazards regression model. Variables used in multivariate analysis were adopted by univariate analysis. The bold terms represent statistical significance (< 0.05).

[A]Patients who developed intrahepatic metastases, or extrahepatic metastases were considered as cases with metastatic potential.

Abbreviations: HCC, hepatocellular carcinoma; HBsAg, hepatitis B surface antigen; ALT, alanine aminotransferase; AFP, α-fetoprotein; TNM, tumor-node-metastasis; OS, overall survival; TR, tumor recurrence; HR, hazard ratio; CI, confidence interval; n.a., not adopted.

Supplemental Table 2. Association of the density of intratumoral $CD68^+CA12^+$ cells with clinicopathological characteristics of 72 patients with HCC.

Variables		Intratumoral $CD68^+CA12^+$ cells		
		Low	High	*P* value
Age, years	≤50	16	21	0.349
	>50	19	16	
Gender	Male	29	31	0.916
	Female	6	6	
HBsAg	Negative	1	1	0.968
	Positive	34	36	
Cirrhosis	Absent	14	12	0.504
	Present	21	25	
ALT, U/L	≤40	19	17	0.479
	>40	16	20	
AFP, ng/ml	≤25	12	10	0.504
	>25	23	27	
Tumor size, cm	≤5	21	14	0.060
	>5	14	23	
Tumor multiplicity	Solitary	30	25	0.070
	Multiple	5	12	
Metastatic potential [A]	No	31	25	**0.032**
	Yes	4	12	
TNM stage	I	29	23	**0.050**
	II + III	6	14	
Tumor differentiation	I + II	20	21	0.974
	III + IV	15	16	

P value was analyzed by Chi-square test. The bold terms represent statistical significance (< 0.05).

[A]Patients who developed intrahepatic metastases, or extrahepatic metastases were considered as cases with metastatic potential.

Abbreviations: HCC, Hepatocellular carcinoma; HBsAg, hepatitis B surface antigen; ALT, alanine aminotransferase; AFP, α-fetoprotein; TNM, tumor-node-metastasis.

Supplemental Table 3. Association of the mRNA expression of CCL8 in tumor-associated macrophages with clinicopathological characteristics of 39 patients with HCC.

Variables		Expression of CCL8 in TAMs		
		Low	High	*P* value
Age, years	≤52	9	11	0.634
	>52	10	9	
Gender	Male	17	15	0.239
	Female	2	5	
HBsAg	Negative	6	4	0.408
	Positive	13	16	
Cirrhosis	Absent	6	4	0.408
	Present	13	16	
ALT, U/L	≤40	13	8	0.075
	>40	6	12	
AFP, ng/ml	≤25	11	6	0.079
	>25	8	14	
Tumor size, cm	≤5	6	2	0.095
	>5	13	18	
Tumor multiplicity	Solitary	13	16	0.408
	Multiple	6	4	
Metastatic potential [A]	No	15	6	**0.002**
	Yes	4	14	
TNM stage	I	6	8	0.584
	II + III	13	12	
Tumor differentiation	I + II	9	12	0.429
	III + IV	10	8	

P value was analyzed by Chi-square test. The bold terms represent statistical significance (< 0.05).

[A]Patients who developed intrahepatic metastases, or extrahepatic metastases were considered as cases with metastatic potential.

Abbreviations: HCC, Hepatocellular carcinoma; HBsAg, hepatitis B surface antigen; ALT, alanine aminotransferase; AFP, α-fetoprotein; TNM, tumor-node-metastasis; TAM, tumor-associated macrophage.

Supplemental Table 4. Clinical characteristics of HCC patients.

Patients characteristics	Cohort 1	Cohort 2
No. of patients	72	49
Age, years (median, range)	50, 23-78	48, 31-71
Gender (male/female)	60/12	42/7
HBsAg (negative/positive)	2/70	10/39
Cirrhosis (absent/present)	26/46	10/39
ALT, U/L (median, range)	40, 8-348	39, 9-1401.3
AFP, ng/ml (≤25/>25)	22/50	17/32
Tumor size, cm (≤5/>5)	35/37	14/35
Tumor multiplicity (solitary/multiple)	55/17	35/14
Vascular invasion (absent/present)	65/7	20/29
Metastatic potential [A] (no/yes)	56/16	29/20
TNM stage (I/II+III)	52/20	14/35
Tumor differentiation (I+II/III+IV)	41/31	24/25

[A]Patients who developed intrahepatic metastases, or extrahepatic metastases were considered as cases with metastatic potential.

Abbreviations: HCC, Hepatocellular carcinoma; HBsAg, hepatitis B surface antigen; ALT, alanine aminotransferase; AFP, α-fetoprotein; TNM, tumor-node-metastasis.

Note: Samples from patients in Cohort 1 were used in Figure 1F, Supplemental Table 1 and Supplemental Table 2; Samples from patients in Cohort 2 were used in Figure 1, A-E, Figure 2A and B, Figure 5, A-C, Figure 5, I-J, Figure 6, H-I, Supplemental Figure 1, Supplemental Figure 2, A-C, Supplemental Figure 3, A-E and Supplemental Table 3.

Supplemental Table 5. Sequences of primers and siRNAs.

Genes		Sequences
Human *CA12* (qPCR)	Forward	TTGGCATCTGTATTGTGGTGGTGG
	Reverse	CAGCTTTGAATTCCTGCTGCTTGG
Human *CA1* (qPCR)	Forward	TTGAGGACAACGATAACCGATCA
	Reverse	CTACGTGAAGCTCGGCAGAAT
Human *CA2* (qPCR)	Forward	ATCGACACTCATACAGCCAAGT
	Reverse	AAAGCATGACCATTGTTGAGGA
Human *CA3* (qPCR)	Forward	AAACCAGTCGCCCGTTGAG
	Reverse	CCACCATCATAAGACACAGACCA
Human *CA4* (qPCR)	Forward	TGGTCCGACTTGCCATATAAGG
	Reverse	CTCTTTCACATTCCTCGATGTCC
Human *CA5A* (qPCR)	Forward	GTGCATGGCAAACCAGCAATA
	Reverse	CCGCTTCATAGGAGACCCTG
Human *CA5B* (qPCR)	Forward	TATGATCCCGGCTTAAAACCAC
	Reverse	GGTAGTTGTGTTCCAGGGGTC
Human *CA6* (qPCR)	Forward	TTTGTGCTGGCAGATTTTGTCA
	Reverse	CTGCGGTAATCGTTGTGGATG
Human *CA7* (qPCR)	Forward	TGACAGCGATGACCGAACC
	Reverse	ACTTCTTGGCATTCCAGTGAAC
Human *CA8* (qPCR)	Forward	GGAAGTCCAAAACAATACCTTGC
	Reverse	TGGTGAGAGAGCCTTCATACAC
Human *CA9* (qPCR)	Forward	TTTGCCAGAGTTGACGAGGC
	Reverse	GCTCATAGGCACTGTTTTCTTCC
Human *CA10* (qPCR)	Forward	CTTTCTGGGGATTGGTGAACTC
	Reverse	TGTGACTGGTCTCTATGTTGACT
Human *CA11* (qPCR)	Forward	AAGCTCCGGGGAACCTTGTA

	Reverse	ACTGAGTCGGTGGCTGTAAAG
Human *CA13* (qPCR)	Forward	CGAGCACAACGGTCCTATTCA
	Reverse	CTAAGTGGTCGGAGGGAAGAG
Human *CA14* (qPCR)	Forward	ACAATGGCTCGCTCACAACTC
	Reverse	GGGCTCGGTAGTTCTGTACCA
Human *VEGFA* (qPCR)	Forward	CATCTTCAAGCCATCCTGTG
	Reverse	TGCATTCACATTTGTTGTGC
Human *VEGFB* (qPCR)	Forward	GAGATGTCCCTGGAAGAACACA
	Reverse	GAGTGGGATGGGTGATGTCAG
Human *PDGFB* (qPCR)	Forward	CTCGATCCGCTCCTTTGATGA
	Reverse	CGTTGGTGCGGTCTATGAG
Human *PDGFA* (qPCR)	Forward	GCAAGACCAGGACGGTCATTT
	Reverse	GGCACTTGACACTGCTCGT
Human *OSM* (qPCR)	Forward	GTGAACGGAACAGGTCTC
	Reverse	GAAGGCAGTGACACCATC
Human *MMP9* (qPCR)	Forward	AGACCTGGGCAGATTCCAAAC
	Reverse	CGGCAAGTCTTCCGAGTAGT
Human *GLUT1* (qPCR)	Forward	CTTTGTGGCCTTCTTTGAAGTG
	Reverse	GACCACACAGTTGCTCCACATAC
Human *HIF1A* (qPCR)	Forward	CCATTAGAAAGCAGTTCCGC
	Reverse	TGGGTAGGAGATGGAGATGC
Human *TNFA* (qPCR)	Forward	AAGCCTGTAGCCCATGTTG
	Reverse	TGGTAGGAGACGGCGATG
Human *IL6* (qPCR)	Forward	TCAGCCCTGAGAAAGGAGACA
	Reverse	GATTTTCACCAGGCAAGTCTCC
Human *IL10* (qPCR)	Forward	CAACCTGCCTAACATGCTTC
	Reverse	CCTTGATGTCTGGGTCTTGG

Human *IL1B* (qPCR)	Forward	CGAATCTCCGACCACCACTAC
	Reverse	GATGAAGGGAAAGAAGGTGCTC
Human *CCL8* (qPCR)	Forward	CATTGTTCTCCCTCCTACCTGTC
	Reverse	AGCACTGATTGCCAAAGAATACC
Human *CDH1* (qPCR)	Forward	CCGCTGGCGTCTGTA GGAAGG
	Reverse	GGCTCTTTGACCACCGCTCTCC
Human *VIM* (qPCR)	Forward	GAGAACTTTGCCGTTGAAGC
	Reverse	TCCAGCAGCTTCCTGTAGGT
Human *ACTB* (qPCR)	Forward	GGATGCAGAAGGAGATCACT
	Reverse	CGATCCACACGGAGTACTTG
Mouse *Ccl8* (qPCR)	Forward	TCTACGCAGTGCTTCTTTGCC
	Reverse	AAGGGGGATCTTCAGCTTTAGTA
Mouse *Vim* (qPCR)	Forward	CGTCCACACGCACCTACAG
	Reverse	GGGGGATGAGGAATAGAGGCT
Mouse *Cdh1* (qPCR)	Forward	GAAGACGCTGAGCATGTGAA
	Reverse	CAGGACCAGGAGAAGAGTGC
Mouse *Actb* (qPCR)	Forward	CCAGGTCATCACTATTGGCAAC
	Reverse	TACGGATGTCAACGTCACAC
Human *CA12* #1 (siRNA)	Sense	GCAAGUCUGUACUGCGGCAdTdT
	Antisense	UGCCGCAGUACAGACUUGCdAdC
Human *CA12* #2 (siRNA)	Sense	GGACAACCCUCAUGACGAAdTdT
	Antisense	UUCGUCAUGAGGGUUGUCCdUdU
Human *HIF1A* (siRNA)	Sense	CUGAUGACCAGCAACUUGAdTdT
	Antisense	UCAAGUUGCUGGUCAUCAGdTdT
Negative control (siRNA)	Sense	UUCUCCGAACGUGUCACGUdTdT
	Antisense	ACGUGACACGUUCGGAGAAdTdT

Supplemental Table 6. Antibodies used in studies.

Name	Supplier	Cat no.	Clone no.
PE/Cyanine7-conjugated anti-mouse CD11b	BioLegend	101215	M1/70
BV421-conjugated anti-mouse Ly6C	BioLegend	128031	HK1.4
BV570-conjugated anti-mouse CD45	BioLegend	103136	30-F11
APC-conjugated anti-mouse F4/80	BioLegend	123116	BM8
PE-conjugated anti-mouse CD204	BioLegend	154709	1F8C33
APC-conjugated anti-mouse CD206	BioLegend	141707	C068C2
BV421-conjugated anti-mouse B220	BioLegend	103251	RA3-6B2
AF700-conjugated anti-mouse CD11b	BioLegend	101222	M1/70
PE-conjugated anti-mouse CD3	BioLegend	100206	17A2
APC-conjugated anti-mouse Gr1	BioLegend	108411	RB6-8C5
PE/Cyanine7-conjugated anti-mouse F4/80	BioLegend	123113	BM8
Alexa Fluor 488 donkey anti-rabbit IgG	Thermo Fisher Scientific	A-21206	polyclonal
FITC-conjugated anti-mouse Gr-1	eBioscience	11-5931-85	RB6-8C5
eFluor 450-conjugated anti-mouse CD8	eBioscience	48-0081-82	53-6.7
PE-conjugated anti-human CD80	eBioscience	12-0809-42	2D10.4
FITC-conjugated anti-mouse Gr1	eBioscience	11-5931-85	RB6-8C5
PE-CF594-conjugated anti-mouse Ly6G	BD Biosciences	562700	1A8
AF700-conjugated anti-human CD14	BD Biosciences	557923	M5E2
PE-conjugated anti-human PD-L1	BD Biosciences	557924	MIH1
APC-conjugated anti-human CD86	BD Biosciences	555660	2331(FUN-1)
BV421-conjugated anti-human HLA-DR	BD Biosciences	562804	G46-6
Mouse anti-human HIF-1α	BD Biosciences	610958	54/HIF-1α
Mouse anti-human CD206	R&D System	MAB25341	685645
Human TNFα neutralizing antibody	R&D Systems	MAB610	28401
Human IL6 neutralizing antibody	R&D Systems	MAB2061	1936
Human IL10 neutralizing antibody	R&D Systems	MAB217	23738
Human IL1β neutralizing antibody	R&D Systems	MAB601	2805
Human CCL8 neutralizing antibody	R&D System	MAB281	35509
Mouse IgG1 Isotype Control	R&D System	MAB002	11711
*InVivo*Mab anti-mouse PD-1 antibody	Bio X Cell	BE0146	RMP1-14
*InVivo*Mab rat IgG2a isotype control antibody	Bio X Cell	BE0089	2A3
*InVivo*Mab anti-mouse CSF1R antibody	Bio X Cell	BE0213	AFS98
Mouse anti-human CD68	DakoCytomation	M0876	PG-M1

Rabbit anti-human GLUT1	Merck Millipore	07-1401	polyclonal
Mouse anti-human CD204	TransGenic	KT022	SRA-E5
Rabbit anti-human CCL8	Abcam	ab9671	polyclonal
Rabbit anti-human CA12	Cell Signaling Technology	5864	D75C6
Rabbit anti-human phospho-p38	Cell Signaling Technology	4511	D3F9
Rabbit anti-human p38	Cell Signaling Technology	9212	polyclonal
Rabbit anti-human phospho-p65	Cell Signaling Technology	3033	93H1
Rabbit anti-human p65	Cell Signaling Technology	8242	D14E12
Rabbit anti-human phospho-IκBα	Cell Signaling Technology	2859	14D4
Mouse anti-human IκBα	Cell Signaling Technology	4814	L35A5
Rabbit anti-human phospho-JNK	Cell Signaling Technology	4668	81E11
Rabbit anti-human JNK	Cell Signaling Technology	9252	polyclonal
Rabbit anti-human phospho-Erk1/2	Cell Signaling Technology	4370	D13.14.4E
Rabbit anti-human Erk1/2	Cell Signaling Technology	4695	137F5
Rabbit anti-human Vimentin	Cell Signaling Technology	5741	D21H3
Rabbit anti-human E-cadherin	Cell Signaling Technology	3195	24E10
Rabbit anti-human N-cadherin	Cell Signaling Technology	13116	D4R1H
Rabbit anti-human SNAI1	Cell Signaling Technology	3879	C15D3
Rabbit anti-human SNAI2	Cell Signaling Technology	9585	C19G7
Rabbit anti-human TWIST1	Cell Signaling Technology	69366	E7E2G
Rabbit anti-mouse F4/80	Cell Signaling Technology	70076	D2S9R
HRP-linked goat anti-rabbit IgG antibodies	Cell Signaling Technology	7074	polyclonal
HRP-linked goat anti-mouse IgG antibodies	Cell Signaling Technology	7076	polyclonal
Rabbit anti-human TWIST2	Signalway Antibody	21669	polyclonal
Mouse anti-human β-actin	Boster	BM0627	AC-15
Mouse anti-human GAPDH	Boster	BM1623	
Rabbit anti-human CD14	SinoBiological	10073-R001	001

Supplemental Table 7. Reagents used in studies.

Name	Supplier	Cat no.
DNase I	Sigma-Aldrich	DN25
Collagenase IV	Sigma-Aldrich	C5138
Hyaluronidase	Sigma-Aldrich	H1136
Collagenase XI	Sigma-Aldrich	C7657
Hepes	Sigma-Aldrich	H4034
MES	Sigma-Aldrich	M3671
2-Deoxy-D-glucose (2DG)	Sigma-Aldrich	D8375
3PO	Sigma-Aldrich	SML1343
Acetazolamide	Sigma-Aldrich	A6011
SB202190	Sigma-Aldrich	S7067
$GdCl_3$	Sigma-Aldrich	439770
L-lactic acid	Sigma-Aldrich	L6402
DMOG	Sigma-Aldrich	D3695
ML-265 (TEPP-46)	Cayman Chemical	13942
DMSO	Merck Millipore	317275
Echinomycin	Merck Millipore	330175
Recombinant human TNFα	R&D Systems	210-TA-020
Recombinant human IL6	R&D Systems	206-IL-010
Recombinant human IL10	R&D Systems	217-IL-005
Recombinant human IL1β	R&D Systems	201-LB-005
Recombinant human CCL8	R&D System	281-CP-010
Cultrex Basement Membrane Extract	R&D Systems	3432-005-01
Penicillin	GENVIEW	AP231
Streptomycin.	GENVIEW	AS325
FBS	Gibco	10099-141
BSA	Biofroxx	4240GR250
Triton X-100	Aladdin	T109027
DAPI	Roche	10236276001
SYTOX Green	Thermo Fisher Scientific	S7020
Human $CD14^+$ MicroBeads	Miltenyi Biotec	130-050-201
ACK lysis buffer	TBD Science	NH4CL2009
Trizol reagent	Thermo Fisher Scientific	AM9738
RPMI 1640 medium	Thermo Fisher Scientific	C11875500BT

DMEM	Thermo Fisher Scientific	C11995500BT
$HCO3^-$-free DMEM	Sigma-Aldrich	D7777
5X All-In-One RT MasterMix	abm	G492
SYBR Green Real-Time PCR Mix	TOYOBO	QPS-201
Tyramide signal amplification kit	Panovue	PPK007100100
Lactate assay kit	eton bioscience	1200011002
P3 primary cell 4D-Nucleofector X kit	Lonza	V4XP-3024
Apoptosis analysis kit	eBioscience	88-8005-72
Human TNFα ELISA kit	eBioscience	88-7346-86
Human IL6 ELISA kit	eBioscience	88-7066-88
Human IL10 ELISA kit	eBioscience	88-7106-88
Human IL1β ELISA kit	eBioscience	88-7261-88
Human CCL8 ELISA kit	BioLegend	442204
Brefeldin A	BioLegend	420601
Clodronate liposome and control liposome	FormuMax	F700101C-NC-10
